# Monocyte-derived Galectin-9 and PD-L1 differentially impair adaptive and innate immune response in chronic HBV infection and their expression remain unaltered after antiviral therapy

**DOI:** 10.3389/fimmu.2024.1474853

**Published:** 2024-10-09

**Authors:** Debangana Dey, Satabdi Biswas, Sourina Pal, Sarthak Nandi, Najma Khatun, Rambha Jha, Bidhan Chandra Chakraborty, Ayana Baidya, Ranajoy Ghosh, Soma Banerjee, SK Mahiuddin Ahammed, Abhijit Chowdhury, Simanti Datta

**Affiliations:** ^1^ Centre for Liver Research, School of Digestive and Liver Diseases, Institute of Post Graduate Medical Education and Research, Kolkata, India; ^2^ Multidisciplinary Research Unit, Institute of Post Graduate Medical Education and Research, Kolkata, India; ^3^ Division of Pathology, School of Digestive and Liver Diseases, Institute of Post Graduate Medical Education and Research, Kolkata, India; ^4^ Department of Hepatology, School of Digestive and Liver Diseases, Institute of Post Graduate Medical Education and Research, Kolkata, India

**Keywords:** immune checkpoint molecules, chronically HBV-infected patients, host and viral factors, immunosuppression, tenofovir

## Abstract

**Introduction:**

Patients with chronic HBV infection (CHI) exhibit defective anti-viral immune-response whose underlying causes still remain unclear. Monocytes act as immune sentinels for pathogens and can regulate immunity via interaction with other immune-cells, apart from differentiating into macrophages. Immune-checkpoint molecules (ICMs) expressed by immune-cells, including monocytes are known to negatively regulate immune-responses. Here, we evaluated the expression of ICMs, namely, Gal-9, PD-L1, and CTLA-4 on monocytes in different phases of CHI, identified the viral and the host factors causing their aberrant expression and investigated their impact during interaction of monocytes with T-cells, B-cells and NK-cells and also on monocyte to macrophage differentiation. Influence of Tenofovir therapy on the expression of monocytic ICMs was also studied.

**Methods:**

Collection of blood and liver-tissue samples from HBV infected patients and controls, flow-cytometry, cell sorting, cell culture and immune-fluorescence were performed for this study.

**Results:**

Gal-9^+^ and PD-L1^+^-monocytes were significantly increased in HBeAg-positive as well as HBeAg-negative chronic hepatitis B (CHB) patients than healthy controls (HC). In immune-tolerant (IT) subjects, only Gal-9^+^-monocytes and in inactive carriers (IC), PD-L1^+^-monocytes were higher than HC while CTLA-4^+^-monocytes remained comparable among groups. High serum Hepatitis B surface antigen (HBsAg) concentration in CHB as well as IT and TNF-α in CHB triggered monocytic Gal-9-expression whereas, PD-L1 was induced by elevated TNF-α and IL-4 in CHB and IL-1β in CHB and IC. Purified monocytes from CHB and IT having high Gal-9 expression led to expansion of CD4^+^CD25^+^FOXP3^+^-Tregs, CD19^+^IL-10^+^-Bregs and CD19^+^CD27^-^CD21^–^atypical memory B-cells and these monocytes also preferentially differentiated into M2-macrophages. These phenomena were reversed by anti-Gal-9-antibody. Parallelly, PD-L1^+^-monocytes in CHB and IC reduced IL-2/IFN-γ and IL-6 production by HBV-specific T- and B-cells respectively, which were restored by anti-PD-L1-antibody. Both Gal-9^+^- and PD-L1^+^-monocytes caused decline in IFN-γ^+^-NK-cells but enhanced IL-10-expressing HBV-specific-T-cells and NK-cells. Increased intrahepatic CD14^+^Gal-9^+^ and CD14^+^PD-L1^+^-monocytes were noted in CHB patients than HC. One-year tenofovir therapy failed to reduce monocytic Gal-9 and PD-L1 along with the levels of HBsAg, TNF-α, IL-1β and IL-4.

**Conclusions:**

Monocytic Gal-9 and PD-L1, expressed heterogeneously in different phases of CHI, exert diverse inhibitory effects on immune-responses and their therapeutic targeting could boost anti-HBV immunity.

## Introduction

The innate and adaptive arms of immune system, upon activation can eliminate invading microbes as well as damaged/abnormal cells and provide crucial protection to host. However, under homeostatic conditions, a network of immunosuppressive pathways comprising of receptors and ligands known as immune checkpoint molecules (ICMs) counterbalance the activation process by modulating the duration and amplitude of immune responses to minimize the collateral damage to healthy cells (1). The ICMs expressed by a variety of immune-cells viz. T-cells, B-cells, monocytes, NK-cells, upon binding with their partner proteins on other cells transduce negative signals and inhibit the immune effector functions. Some important immune checkpoint receptor-ligand pairs include PD-1 and its ligand PD-L1, CTLA-4 and its receptor B7-molecules CD80/CD86 and TIM-3 and its ligand Gal-9 (2). Different studies have documented that cancer progression is associated with increased expression of multiple inhibitory receptors, including, PD-1/TIM-3 on tumor-specific T-cells, which cause functional exhaustion of these cells, thereby hindering tumor elimination (2). Hence, immune checkpoint blocking therapy aiming to re-invigorate the T-cell responses are gradually replacing chemotherapy as the cornerstone of treatment of malignant tumours. The development of chronic viral infection is also promoted by different immune subversion mechanisms that include activation of immune checkpoint pathways (1). Hence, understanding the ICM functions and their subsequent targeting would help in enhancing the anti-viral immune responses and effective control of infection. Chronic HBV infection (CHI) represents a major global health problem and is associated with a diverse spectrum of disease and natural history ranging from minimal hepatic lesions to severe liver diseases, such as cirrhosis and hepatocellular carcinoma (HCC) (3). The natural history of CHI has been divided into four distinct phases namely, (1) immune-tolerance (IT) [recently designated as hepatitis B e-antigen (HBeAg)-positive chronic HBV infection], (2) HBeAg-positive chronic hepatitis B (EP-CHB), (3) inactive carrier (IC) (renamed as HBeAg-negative chronic HBV infection) and (4) HBeAg-negative chronic hepatitis B (EN-CHB) and each phase reflects varying levels of host immune activation in response to HBV that mediate both liver injury and virus control (4). Current antiviral therapy with nucleotide/nucleoside analogs can suppress HBV replication but fail to eliminate the virus and the development of new therapeutic strategies remains a key unmet medical need (4). Moreover, marked impairment in host immune responses had been reported during CHI. HBV-specific T-cells of CHB patients had been found to overexpress multiple ICMs such as, PD-1, CTLA-4 and TIM-3 (5) while increased Gal-9 expression had been observed in circulating NK-cells (6) contributing to their dysfunction. In chronic HCV infection, monocyte-derived Gal-9 had been shown to enhance NK-cell cytotoxicity, which might be associated with liver injury (7). However, there is limited information on ICMs expressed on monocyte surface during CHI and their effects in shaping the immune response. Monocytes represent a central part of innate immunity and their phenotypic pliability, differentiation ability and capacity to cross-talk with other immune-cells namely, T-cells, B-cells and NK-cells make them crucial players in liver homeostasis and disease pathogenesis. Hence, we sought to investigate the ICM expression pattern on monocytes in various phases of CHI, the factors that regulate their expression and the distinctive immune response they elicit during interaction of monocytes with other important immune cells. Additionally, the effect of antiviral therapy on monocytic ICM in CHB patients was also explored. Understanding the phase-specific role of monocyte-associated ICMs would be useful in defining effective immunotherapeutic strategies for achieving functional cure of CHB.

## Methods

### Study subjects and samples

Treatment-naïve patients with CHI [having hepatitis B surface antigen (HBsAg) positivity for greater than six months] were included in the study from School of Digestive and Liver Diseases (SDLD), I.P.G.M.E.&R., Kolkata, India. All necessary virological, biochemical and clinical assessments were conducted before categorization of these patients into distinct study groups namely, IT, EP-CHB, IC and EN-CHB. IT group included participants having HBeAg-positivity, high HBV-DNA (>10^7^ copies/ml) but normal serum alanine transaminase (ALT) levels (≤40 IU/L) in three consecutive follow-ups within a period of 1 year prior to enrolment and had minimal or no liver necro-inflammation. Both EP-CHB and EN-CHB patients were diagnosed with HBV-DNA>10^4^ copies/ml, ALT>40 IU/L and evidence of active hepatic necro-inflammatory activity, the only difference being presence or absence of detectable HBeAg in their serum. IC were characterized by HBeAg-negativity, HBV-DNA<10^4^ copies/ml and ALT≤ 40 IU/L, both measured on three occasions 3 months apart, with no biochemical, clinical or histological evidence of liver disease.

Patients having co-infection with HIV/HCV/HDV, significant co-morbidities like diabetes mellitus, chronic alcoholism, intravenous drug abuse or evidence of any carcinoma, overt infection or autoimmune disorders were excluded from the study.

In addition, healthy individuals (HC) as evident from their HBsAg negative status, no traces of viral and bacterial infection, normal liver function (having no clinical, biochemical and imaging evidence of liver disease), no history of acute or chronic illness (like diabetes, hypertension) which required medical or surgical intervention in the preceding 6 months and no history of significant alcohol intake were enrolled as a comparison group for the study. For characterizing HBV-specific T- and B-cells, acutely HBV-infected patients who resolved infection (having HBsAg negativity and positive for antibody against hepatitis B core antigen) were recruited as controls. Written informed consents were taken from all participants. All study protocols were approved by the Ethical Review Committee of I.P.G.M.E.&R.

### Assessment of ICMs expressed by monocytes in different study groups

To assess the expression of ICMs, Gal-9, PD-L1 and CTLA-4 on HLA-DR^+^CD14^+^ total monocytes, CD14^++^CD16^−^ classical monocytes, CD14^++^CD16^+^ intermediate monocytes and CD14^+^CD16^++^ non-classical monocytes, freshly collected EDTA-mixed blood from different groups of treatment-naïve chronically HBV infected patients and HC were incubated with anti-HLA-DR-V500, anti-CD14-FITC, anti-CD16-PECY7, anti-Gal-9-PerCP, anti-PD-L1-PE and anti-CTLA-4-APC followed by treatment with FACS Lysing Solution (BD Pharmingen) for erythrocyte lysis. Cells were then washed and acquired on FACSVerse (BD Biosciences). In each set, fluorescence minus one (FMO) control was used to minimize background noise. The flow cytometry data analysis was performed using FCS Express *De Novo* software.

### Quantification of Hepatitis B surface antigen and cytokines in serum

Serum HBsAg and different cytokines in study subjects were quantified using Abbott Architect i1000sr platform and BD CBA Human Th1/Th2/Th17 Cytokine Kit and IL-1 beta ELISA Kit.

### Treatment of sorted monocytes or PBMCs with recombinant HBsAg, rTNF-α, rIL-4 or rIL-1β

From healthy individuals, peripheral blood mononuclear cells (PBMCs) were isolated from freshly collected EDTA-mixed blood using lymphocyte separation media (LSM-1077) (HiMedia) by density gradient centrifugation. The monocytes were sorted from PBMCs using CD14 microbeads and magnetic separation columns (Miltenyi Biotech). 2×10^6^ monocytes were cultured for 48 hours in presence or absence of recombinant HBsAg (20μg/ml) (Abcam) or β-galactosidase (20μg/ml) (Sigma-Aldrich). The cells were then harvested followed by staining with anti-CD14-FITC, anti-HLA-DR-BV421, anti-CD16-PE-Cy7, anti-Gal-9-PerCP and anti-PD-L1-APC followed by washing and acquisition in flow cytometer.

Additionally, in separate sets of experiment, the PBMCs of healthy individuals were treated with rTNF-α (50 ng/ml), rIL-4 (25 ng/ml) or rIL-1β (5 ng/ml) for 3 days or kept untreated. The cells were harvested at day 3, followed by staining with antibodies against HLA-DR, CD14, CD16, Gal-9 and PD-L1. The cells were then washed and acquired in flow cytometer.

### Characterization of different adaptive and innate immune cells

To determine the cytokine production of HBV-specific T-cells, PBMCs were isolated from chronically HBV infected patients belonging to different disease phases and the acutely HBV infected patients who resolved infection, as these patients were supposed to exhibit HBV-specific T-cell response. Briefly, 2x10^6^ PBMCs were stimulated with a set of 15-mer peptides overlapping by 10 residues (OLP) spanning genotype D HBV core protein (5μg/ml) (Mimotopes) for 5 days at 37°C. On the 4^th^ day, the cells were restimulated with 5μg/ml OLP along with 1μg/ml Brefeldin A for overnight. At day 5, the cells were harvested and stained with anti-CD3-PE-CY7, anti-CD4-FITC and anti-CD8-PerCP, washed, permeabilized and fixed with BD Cytofix/Cytoperm kit and again stained with anti-IFN-γ-BV421, anti-IL-2-PE and anti-IL-10-APC antibodies followed by acquisition in FACSVerse.

To characterize the regulatory T-cells (Tregs), the PBMCs were stimulated with anti-CD3/anti-CD28 and cultured for 3 days. The cells were harvested at the end of day 3 and incubated with fluorochrome conjugated antibodies against CD4 and CD25 in proper combination and washed. To evaluate the expression of FOXP3 on CD4^+^CD25^+^ T-cells, cells were incubated with FOXP3 Fix/Perm buffer (Biolegend, USA) and subsequently with FOXP3 washing buffer (Biolegend, USA) for fixation and intranuclear permeabilization. The cells were stained with anti-FOXP3-PE and analysed by flow cytometry.

To determine IL-6 production by HBV-core antigen specific B-cells, PBMCs from HBV infected patients were stimulated with rHBcAg (4μg/ml) for 3 days at 37°C. On the 3^rd^ day, the cells were harvested and stained with anti-CD19-FITC, washed, permeabilized and fixed with BD Cytofix/Cytoperm kit and again stained with anti-IL-6-PE antibody followed by acquisition and analysis.

To ascertain the frequencies of atypical memory B (atMB) cells and regulatory B-cells (Bregs), PBMCs of the study subjects were stimulated with rCD40L (1μg/ml) followed by staining with fluorochrome conjugated antibodies against CD19, CD27 and CD21 in proper combination. The cells were then fixed and permeabilized as before, stained with fluorochrome conjugated anti-IL-10 antibody and acquired using flow cytometry.

To characterize the cytokine production of the NK-cells, PBMCs of the study subjects were stimulated by rIL-12 and rIL-18 (5ng/ml) antibodies for 24 hours followed by harvesting of cells, staining with fluorochrome conjugated antibodies against CD3, CD56 and CD16 in proper combination. The cells were then fixed and permeabilized and again stained with anti- IFN-γ-PE and anti-IL-10-APC. The percentages of IFN-γ- and IL-10-expressing CD3^-^CD56^+^CD16^+^ NK-cells were determined.

### Co-culture of CD14^+^monocytes and monocyte-depleted PBMC

CD14^+^-monocytes were sorted from PBMCs of chronically HBV-infected patients, co-cultured with monocyte-depleted PBMC pre-treated with HBV core OLP and anti-CD3/CD28 and the percentages of IFN-γ^+^, IL-2^+^ and IL-10^+^ HBV-specific CD4^+^-/CD8^+^-T-cells and CD4^+^CD25^+^FOXP3^+^-Tregs were evaluated by flow-cytometry as described before. Similar co-culture experiments were performed after pre-treating the monocyte-depleted PBMCs with rHBcAg or rCD40L and the frequencies of IL-6^+^-HBcAg-specific B-cells, atMB-cells and Bregs of chronically HBV-infected patients were determined.

To study the effect of monocytes on the cytokine production by NK-cells, monocytes were cultured with monocyte-depleted PBMCs incubated with rIL-12 and rIL-18 and IFN-γ- and IL-10-expressing NK-cells were analyzed by flow-cytometry. All co-culture experiments were performed in presence or absence of anti-Gal-9- or anti-PD-L1-antibody.

### Differentiation of monocytes to macrophages

To differentiate the monocytes into macrophages, 2×10^7^ PBMCs were allowed to adhere for 5 hours in serum free condition. Then the serum free media was replaced carefully with RPMI medium supplemented with 10% FBS and the cells were cultured in presence of M-CSF (50ng/ml) for 7 days. At day 7, the cells were exposed to fresh medium containing either IFN-γ (20 ng/ml) and LPS (100 ng/ml) or IL-4 (25 ng/ml) for additional 24 hours to terminally polarize them to M1- or M2-macrophages respectively. Next, the cells were washed and stained with anti-HLA-DR-V500, anti-CD14-PerCP and anti-CD68-FITC, followed by fixation and permeabilization and staining either with anti-TNF-α-APC or with anti-IL-10-APC. The macrophages were identified as CD14^+^HLA-DR^+^CD68^+^ cells by flow cytometry. The proportion of TNF-α^+^- and IL-10^+^- cells among the macrophages denoted M1 and M2-macrophages respectively and M1-/M2 macrophage ratio was calculated.

### Immunofluorescence staining

Percutaneous liver biopsy specimens were obtained from selected CHB patients as part of their clinical assessment as deemed necessary by the clinician for studying the liver histopathology. Liver tissues from healthy donors were collected as part of the pre-transplantation assessment for living-donor liver transplantation. Immunohistochemical analysis were performed on stored paraffin embedded tissue blocks of selected CHB patients and healthy donors to study the intrahepatic incidence of Gal-9^+^CD14^+^ and PD-L1^+^CD14^+^ monocytes. Haematoxylin and eosin (H&E) staining was performed with formalin-fixed, paraffin-embedded 6μm liver tissue sections to evaluate the histological status of the liver of study subjects and viewed under light microscope. For immunohistochemical analyses, after deparaffinization, hydration and antigen retrieval, tissue sections were stained with anti-CD14-FITC (1:600 dilution), anti-Gal-9-PerCP (1:700 dilution), anti-PD-L1-APC (1:1000 dilution) followed by washing and cover-mounting with Pro-Long Gold Antifade/DAPI solution. The sections were examined under fluorescence microscope (Leica Thunder Imager, Leica Microsytems, Germany).

### Assessment of virological and immunological parameters following Tenofovir-therapy

Blood samples were collected from 15 CHB patients treated with Tenofovir before initiation (pre-TDF) and after 12 months of therapy (post-TDF) and frequencies of Gal-9^+^-/PD-L1^+^-monocytes and serum HBV-DNA, HBsAg, ALT and cytokine levels were appraised.

### Statistical analysis

Statistical analysis was performed using GraphPad Prism9 software as relevant. *P*< 0.05 was considered statistically significant.

## Results

### Differential monocytic ICM-expression in various phases of CHI

**Figure 1:**
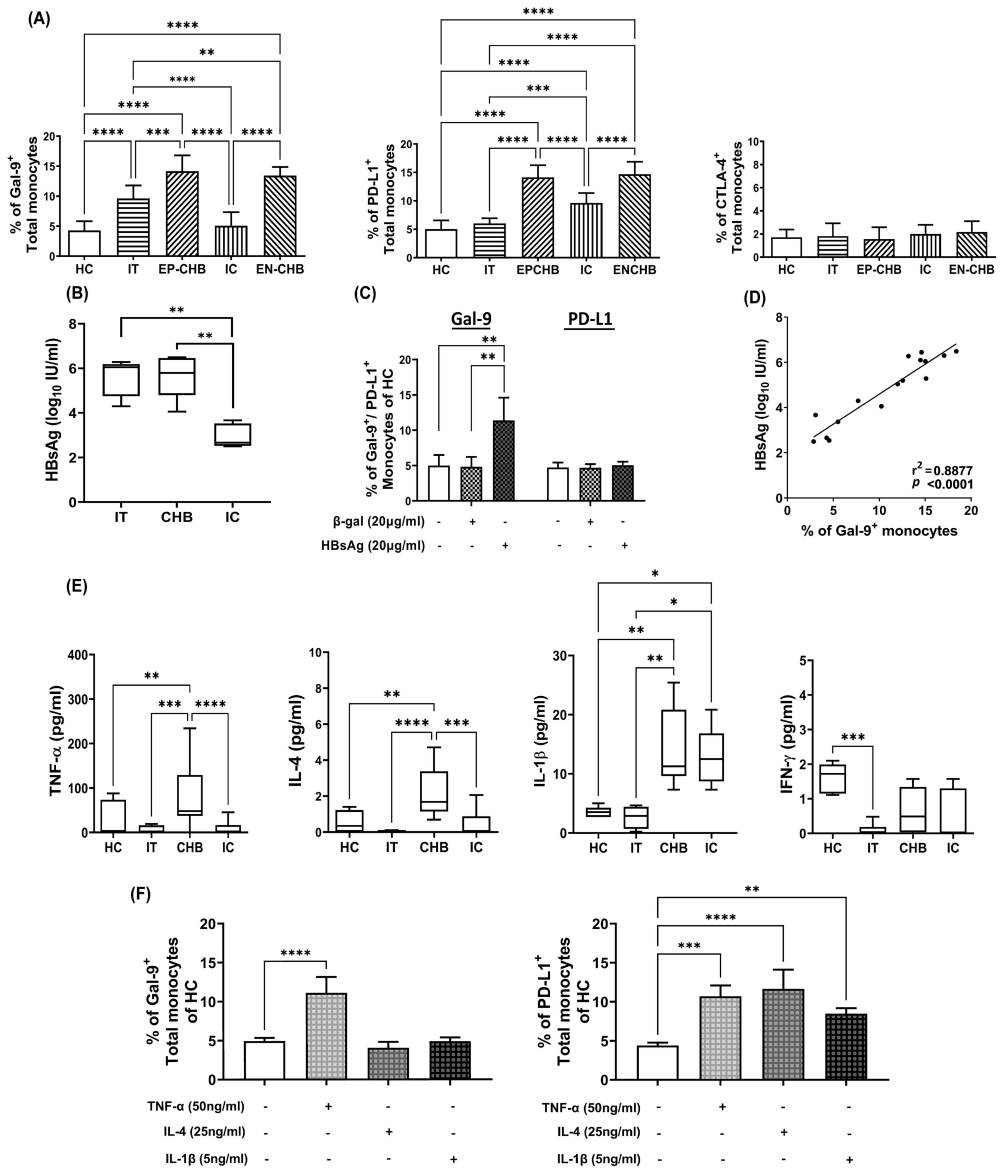
Expression of ICMs on monocytes in chronically HBV-infected patients and its regulation by viral/host factors: Grouped bar diagrams demonstrating percentages of total monocytes expressing **(A)** Gal-9, PD-L1 and CTLA-4 in immune-tolerant (IT) (n=10), HBeAg-positive CHB (EP-CHB) (n=20), inactive carriers (IC) (n=20), HBeAg-negative CHB (EN-CHB) (n=21), and healthy controls (HC) (n=20). **(B)** Serum level of hepatitis B surface antigen (HBsAg) in IT, CHB (both EP-/EN-CHB), and IC. **(C)** The proportion of sorted CD14^+^ monocytes of healthy controls (HC) that expressed Gal-9 and PD-L1 following treatment with recombinant Hepatitis B surface antigen (rHBsAg) (20μg/ml). Untreated cells or β‐galactosidase (β‐gal) (20μg/ml) treated monocytes were kept as control. **(D)** Correlation analysis between percentages of Gal-9 expressing monocytes and level of serum HBsAg in chronically HBV infected patients. **(E)** Serum concentration of TNF-α, IL-4, IL-1β and IFN-γ in IT, CHB, IC, and HC. **(F)** Frequencies of Gal-9 and PD-L1-expressing monocytes in PBMCs of HC treated with recombinant (r) rTNF-α, rIL-4, and rIL-1β. Mean ± SD are presented. Means among groups were compared by one-way ANOVA with Tukey’s multiple comparison or two-way ANOVA as appropriate in **(A, B, D–F)**. *p < 0.05, **p < 0.01, ***p < 0.001 and ****p < 0.0001 Correlation was assessed by linear regression analysis (p < 0.0001) in **(D)**. Mean ± SD of three individual set of experiments is given in **(C)** and **(F)**.

A total of 71 chronically HBV-infected patients were categorised as IT (n=10), EP-CHB (n=20), IC (n=20) and EN-CHB (n=21) based on clinical, serological, virological and histological parameters (**Supplementary Table S1**). We first evaluated the frequencies of total circulating monocytes and their subsets expressing the ICMs, Gal-9, PD-L1 and CTLA-4 in various phases of CHI. The percentages of Gal-9-expressing total-, classical-, intermediate- and non-classical-monocytes were found to be significantly elevated in IT, EP- and EN-CHB compared to IC and HC (**Figure 1A**, **Supplementary Figure S1**). However, Gal-9^+^-monocytes were significantly higher in EP-/EN-CHB patients than IT. In contrast, significant enhancement of PD-L1^+^-total-, classical-, intermediate and non-classical-monocytes was noticed in EP-/EN-CHB and IC relative to IT and HC (**Figure 1A**, **Supplementary Figure S1**). Additionally, CHB patients harbored higher frequency of PD-L1-expressing monocytes than IC. Further in EP-/EN-CHB, dual Gal-9/PD-L1-expression was detected in only ~6% of total monocytes, while these cells were barely perceptible in IT, IC or HC (**Supplementary Figure S2**). On the hand, the proportion of CTLA-4^+^-monocytes was found to be comparable across all study groups. In all cases, Gal-9, PD-L1 and CTLA-4 were expressed predominantly by intermediate- and non-classical-monocytes (**Supplementary Figure S1**).

### HBsAg and cytokine milieu regulate Gal-9/PD-L1-expression on monocytes

It has been reported in different studies that viral antigens may cause aberrant immune responses (8). We next sought to identify the viral antigen that might regulate Gal-9 and PD-L1-expression on monocytes during CHI. Given that HBsAg is the most abundant viral protein in the blood of HBV-infected individuals, we evaluated the serum concentration of HBsAg in chronically HBV-infected patients representing different disease phases. HBsAg level was found to be markedly high in IT and EP-/EN-CHB than IC (**Figure 1B**). Treatment of sorted CD14^+^-monocytes of HC with rHBsAg caused significant augmentation in Gal-9^+^-monocytes but did not induce any change in PD-L1-expressing monocytes relative to controls (**Figure 1C**, **Supplementary Figure S3**), implying that greater prevalence of Gal-9^+^-monocytes in IT and CHB could be attributed to high HBsAg titre in these patients. Moreover, serum HBsAg titre of chronically HBV-infected patients correlated positively with Gal-9^+^-monocytes (**Figure 1D**). Assessment of serum cytokine levels in study subjects indicated significant increases in TNF-α and IL-4 exclusively in EP-/EN-CHB than the other groups, whereas IL-1β was high in both CHB and IC relative to IT and HC (**Figure 1E**). IFN-γ level, however, remained comparable in HC, CHB and IC but low in IT. It was observed that treating PBMC of HC with rTNF-α conferred significant enhancement in the proportions of both Gal-9^+^- and PD-L1^+^-monocytes whereas, rIL-4 and rIL-1β caused marked rise in only PD-L1-expressing monocytes and had little effect on Gal-9 (**Figure 1F**, **Supplementary Figure S4**). Collectively, high concentration of HBsAg and TNF-α could induce monocytic Gal-9 while PD-L1 was stimulated by TNF-α, IL-4 and IL-1β.

### Monocyte-derived Gal-9 and PD-L1 exerted diverse effects on cytokine production by HBV-specific T-cells in different disease phases

**Figure 2:**
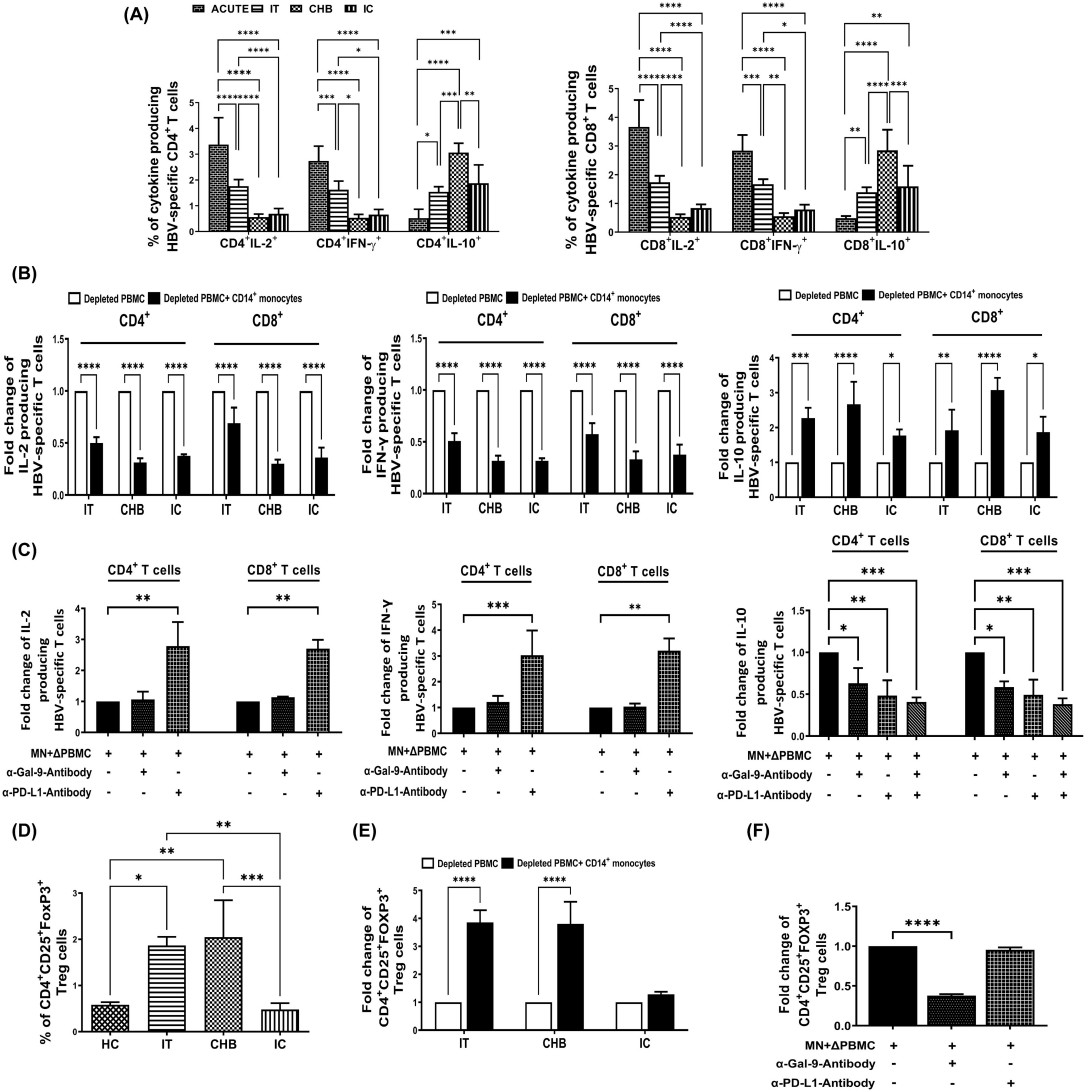
Monocytic Gal-9/PD-L1 differentially regulate cytokine production by HBV-specific T-cells and Treg frequency during CHI. **(A)** Bar diagrams depicting percentages of HBV-specific CD4^+^ and CD8^+^ T cells expressing cytokines IL-2, IFN-γ and IL-10 in acutely HBV infected patients with resolved infection (ACUTE) (n=10), immune-tolerant (IT) (n=10), chronic hepatitis B (CHB) (n=10) and inactive carriers (IC) (n=10). **(B)** Grouped bar diagrams representing fold changes in the frequencies of HBV-specific CD4^+^ and CD8^+^ T-cells expressing IL-2, IFN-γ and IL-10 following co-culture of sorted CD14^+^ monocytes with HBV core peptide stimulated autologous monocyte-depleted PBMCs derived from IT, CHB, and IC. **(C)** Monocytes (MN) sorted from CHB were co-cultured with autologous monocyte depleted PBMCs (ΔPBMC) stimulated with HBV core peptide in absence or presence of anti-Gal-9 (α-Gal-9) or/and anti-PD-L1-antibody (α-PD-L1) and percentages of IL-2, IFN-γ and IL-10 expressing HBV-specific CD4^+^ as well as CD8^+^ T-cells were assessed. **(D)** Grouped bar diagrams demonstrating frequencies of CD4^+^CD25^+^FOXP3^+^ regulatory T-cells (Tregs) in IT (n=10), CHB (n=10), IC (n=10) and healthy controls (HC) (n=10). **(E)** Fold changes in the frequencies of Tregs following co-culture of sorted CD14^+^ monocytes with anti-CD3/anti-CD28 treated autologous monocyte-depleted PBMCs derived from IT, CHB, and IC. **(F)** Assessment of frequency of Tregs following co-culture of Monocytes (MN) sorted from CHB and autologous monocyte depleted PBMCs (ΔPBMC) stimulated with anti-CD3/anti-CD28 in absence or presence of anti-Gal-9 (α-Gal-9) and anti-PD-L1-antibody (α-PD-L1) separately. Comparisons between groups were performed using the one-way ANOVA with p values adjusted by the Tukey’s multiple comparison test in **(A)** and **(D)**. t Tests were performed for comparing paired groups and Mean ± SD of three individual set of experiments in each study group is given in **(B, C, E, F)**. *p < 0.05, **p < 0.01, ***p < 0.001, ****p < 0.0001.

Previous research had demonstrated impaired effector functions of HBV-specific T-cells in CHB patients (5). To explore whether monocyte-derived ICMs contribute to this defect, we first studied the cytokine production by HBV-specific T-cells in different clinical stages. High frequencies of IL-2- and IFN-γ-producing HBV-specific CD4^+^-/CD8^+^-T-cells were noted in patients with resolved HBV infection followed by IT while such cells were markedly low in CHB and IC (**Figure 2A**). Conversely, IL-10 was minimally produced by HBV-specific T-cells of patients with resolved infection whereas it was most pronounced in CHB patients followed by both IT and IC (**Figure 2A**).

It was observed that co-culture of sorted CD14^+^-monocytes from CHB patients with autologous monocyte-depleted PBMCs stimulated with HBV core peptides, led to substantial reduction in the percentages of HBV-specific IL-2^+^-CD4^+^/CD8^+^ T-cells by ~3.2-fold, whereas the corresponding decline was ~2.8-fold in the presence of monocytes sorted from IC (**Figure 2B**). On the other hand, monocytes from IT restricted the IL-2 secretion of CD4^+^-T-cells by ~1.9-fold and that of CD8^+^ T-cells by ~1.6-fold. In case of IFN-γ, monocytes isolated from IT, CHB, and IC resulted in marked diminution in IFN-γ-expressing virus-specific CD4^+^-T-cells by almost 1.9-fold, 3.2-fold, and 3.1-fold, respectively and that of IFN-γ^+^CD8^+^-T-cells by about 1.7-fold, 3.1-fold, and 2.8-fold (**Figure 2B**). Thus, the monocyte-mediated suppression of IFN-γ secretion by HBV-specific T-cells was most profound in CHB and IC followed by IT. With regard to IL-10, monocytes of IT, CHB, and IC caused significant increase in IL-10 production from HBV-specific CD4^+^-T-cells by about 2.3-fold, 2.7-fold, and 1.7-fold respectively and from HBV-specific CD8^+^-T-cells by almost 2.0-fold, 3.0-fold, and 1.8-fold (**Figure 2B**).

We further studied the individual contribution of monocytic Gal-9 and PD-L1 in inhibiting IL-2 and IFN-γ and in augmenting IL-10 production by HBV-specific T-cells. Treating monocytes of CHB patients with anti-PD-L1-antibody during co-culture with target PBMCs led to significant increase in IL-2 production by both CD4^+^- and CD8^+^-HBV-specific T-cells by ~2.8-fold, and IFN-γ production by ~3.2-fold with respect to control setup without inhibitor (**Figure 2C**, **Supplementary Figures S5A, B**). However, no change in IL-2 and IFN-γ production by HBV-specific T-cells was detected when anti-Gal-9-antibody was added (**Figure 2C**, **Supplementary Figures S5A, B**). In contrast, individual treatment of anti-PD-L1- and anti-Gal-9-antibody could markedly diminish IL-10-expressing virus-specific CD4^+^-/CD8^+^-T-cells by about ~2-fold and ~1.7-fold respectively, whereas addition of anti-Gal-9 and anti-PD-L1-antibody together resulted in ~2.5-fold reduction in IL-10-expressing virus-specific CD4^+^-/CD8^+^-T-cells (**Figure 2C**, **Supplementary Figure S5C**). Taken together, our results indicated that monocyte-associated PD-L1 could impair both IL-2 and IFN-γ secretion by HBV-specific T-cells whereas Gal-9 exerted minimal effects on these two cytokines. However, both PD-L1 and Gal-9 could enhance IL-10 production by T-cells.

### Gal-9 expressed by monocytes promoted Treg generation in IT and CHB patients

CHB patients had been reported to harbour high frequency of CD4^+^CD25^+^FOXP3^+^-Tregs (9) and we explored whether monocyte associated ICMs could drive Treg induction in CHI. We noted high percentages of Tregs in IT and CHB as compared to IC/HC (**Figure 2D**). Co-culture of CD14^+^-monocytes sorted from both IT and CHB patients with autologous monocyte-depleted PBMC revealed ~3.8-fold higher incidence of Treg whereas in presence of monocytes sorted from IC, the percentages of Treg were increased by only ~1.3-fold (**Figure 2E**). Moreover, the expansion of Treg in CHB was significantly reversed upon treating monocytes with anti-Gal-9-antibody but remained unaltered upon anti-PD-L1 treatment (**Figure 2F**, **Supplementary Figure S6**), signifying an essential role of Gal-9 in Treg generation.

### Monocytic PD-L1 attenuate cytokine production by HBcAg-specific B-cells in CHB/IC

**Figure 3:**
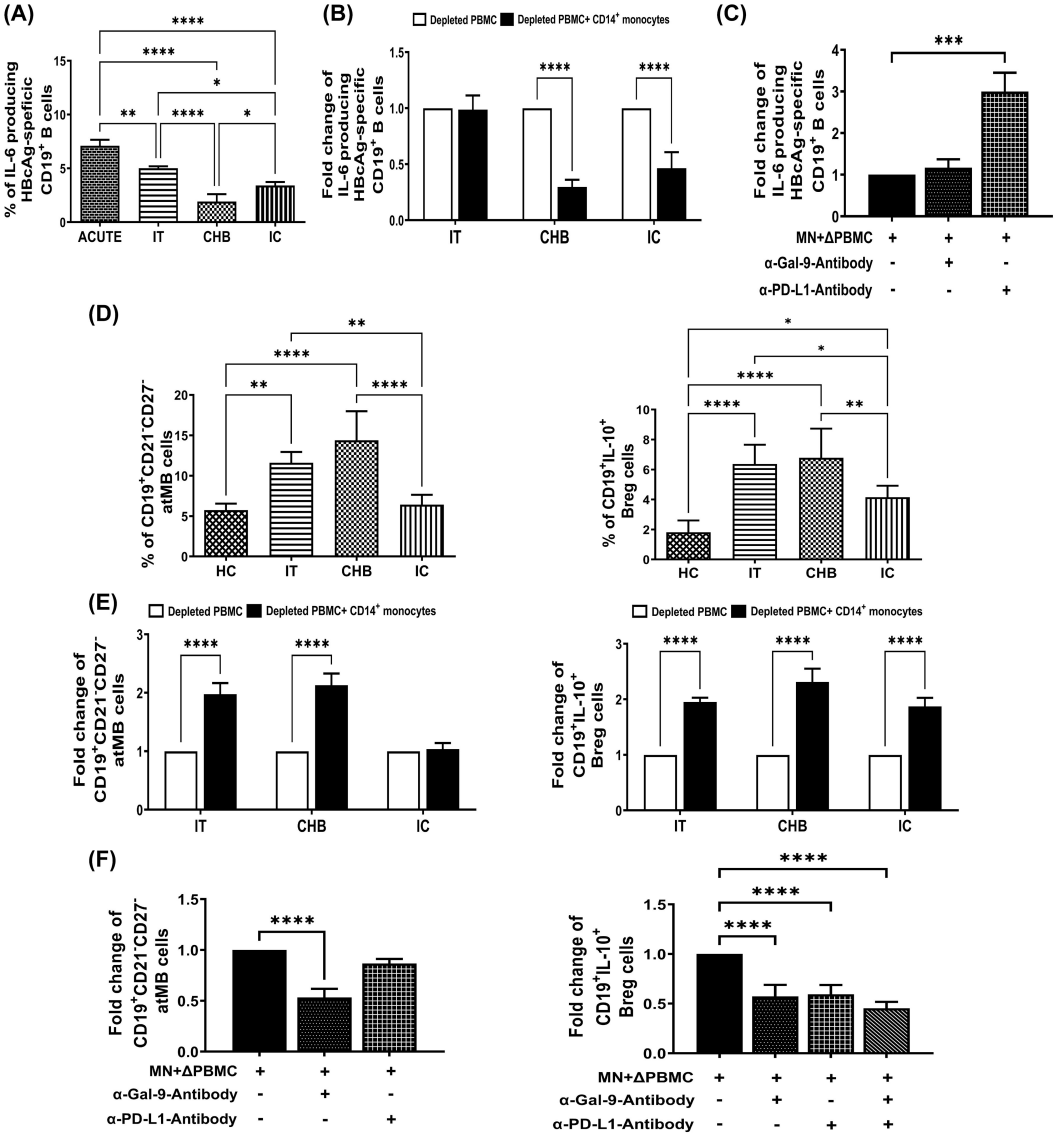
Monocyte-associated Gal-9/PD-L1 regulate frequencies of IL-6^+^-HBcAg-specific B cells, atMB-cells and Bregs during CHI. **(A)** Bar diagrams depicting percentages of HBV-core antigen specific CD19^+^ B-cells expressing cytokines IL-6 in acutely HBV infected patients with resolved infection (ACUTE) (n=10), immune-tolerant (IT) (n=10), chronic hepatitis B (CHB) (n=10) and inactive carriers (IC) (n=10). **(B)** Grouped bar diagram representing fold changes in the frequencies of CD19^+^IL-6^+^ HBcAg-specific B-cells following co-culture of sorted CD14^+^ monocytes with rHBcAg-stimulated autologous monocyte-depleted PBMCs derived from IT, CHB, and IC. Mean ± SD of three individual set of experiments is given. **(C)** Evaluation of IL-6 expressing HBcAg-specific CD19^+^ B cells following co-culture of Monocytes (MN) sorted from CHB and autologous monocyte depleted PBMCs (ΔPBMC) stimulated with rHBcAg in absence or presence of anti-Gal-9 (α-Gal-9) and anti-PD-L1-antibody (α-PD-L1). **(D)** Grouped bar diagrams representing pooled data of frequencies of CD19^+^CD21^-^CD27^-^ atypical memory B (atMB) cells (left panel) and CD19^+^IL-10^+^ regulatory B (Breg) (right panel) cells in IT, CHB IC and healthy controls (HC). **(E)** Bar diagrams representing fold changes in the frequencies of atMB cells (left panel) and Bregs (right panel) following co-culture of sorted CD14^+^ monocytes with autologous rCD40L-stimulated monocyte-depleted PBMCs derived from IT, CHB and IC. **(F)** Determination of frequencies of atMB cells (left panel) and Breg cells (right panel) following co-culture of Monocytes (MN) sorted from CHB and autologous monocyte depleted PBMCs (ΔPBMC) stimulated with rCD40L in absence or presence of anti-Gal-9 (α-Gal-9) and anti-PD-L1-antibody (α-PD-L1). Comparisons between groups were performed using the one-way ANOVA with p values adjusted by the Tukey’s multiple comparison test in **(A)** and **(D)**. t Tests were performed for comparing paired groups and Mean ± SD of three individual set of experiments in each study group is given in **(B, C, E, F)**. *p < 0.05, **p < 0.01, ***p < 0.001, ****p < 0.0001.

B-cells are important source of cytokines in viral infections (10), among which IL-6 has potent antiviral activity. It was observed that the percentage of IL-6-expressing HBcAg-specific B-cells were greater in patients with resolved HBV infection followed by IT but significantly less in CHB and also in IC (**Figure 3A**). We next examined the contribution of monocytes towards change in IL-6 production by these B-cells. Co-culture of purified monocytes with monocyte-depleted PBMC (having B-cells) incubated with rHBcAg, depicted that in IT, the monocytes exerted no significant effect on IL-6 secretion by HBcAg-specific B-cells. In striking contrast, monocytes of CHB and IC induced ~3.5-fold and ~2.3-fold decrease respectively in the frequencies of IL-6^+^-HBcAg-specific B-cells (**Figure 3B**, **Supplementary Figure S7A**). Since increased PD-L1 was a prominent feature of monocytes of CHB and IC but not IT, we tested the potential of PD-1 blockade to rescue B-cell responses in CHB. Addition of anti-PD-L1-antibody during co-culture increased IL-6 release by HBcAg-specific B-cells by ~3-fold whereas cytokine production was not altered in presence of anti-Gal-9-antibody (**Figure 3C**, **Supplementary Figure S7A**).

### Monocyte-derived Gal-9 potentiates atMB-cell amplification while Gal-9 and PD-L1 enhance Bregs

Altered distribution of B-cell subsets, including expansion of atMB-cells and Bregs had been seen in CHB patients (11, 12), both of which can contribute to immune dysfunction. We also noted that CD19^+^CD21^-^CD27^-^ atMB-cells were particularly enriched in IT and CHB compared to IC and HC. In contrast, Bregs were markedly elevated in all phases, IT/CHB/IC relative to HC, although CHB and IT harboured higher proportions of Bregs than IC (**Figure 3D**). Co-culture experiments of monocytes with target PBMC in presence/absence of anti-Gal-9- or anti-PD-L1-antibody depicted increased atMB-cells in presence of monocytes of IT and CHB but not IC and this was inhibited by treatment with only anti-Gal-9-antibody (**Figures 3E, F**, **Supplementary Figure S7B**). On the other hand, monocyte-mediated accumulation of Breg was abrogated by anti-Gal-9- and anti-PD-L1-antibody when they are added separately or in combination (**Figures 3E, F**, **Supplementary Figure S7C**). These findings strongly suggest monocytic Gal-9-associated induction of atMB-cells while both Gal-9 and PD-L1 triggered Breg generation.

### Gal-9 and PD-L1 expressed by monocytes mediate NK-cell dysfunction in different phases of CHI

**Figure 4:**
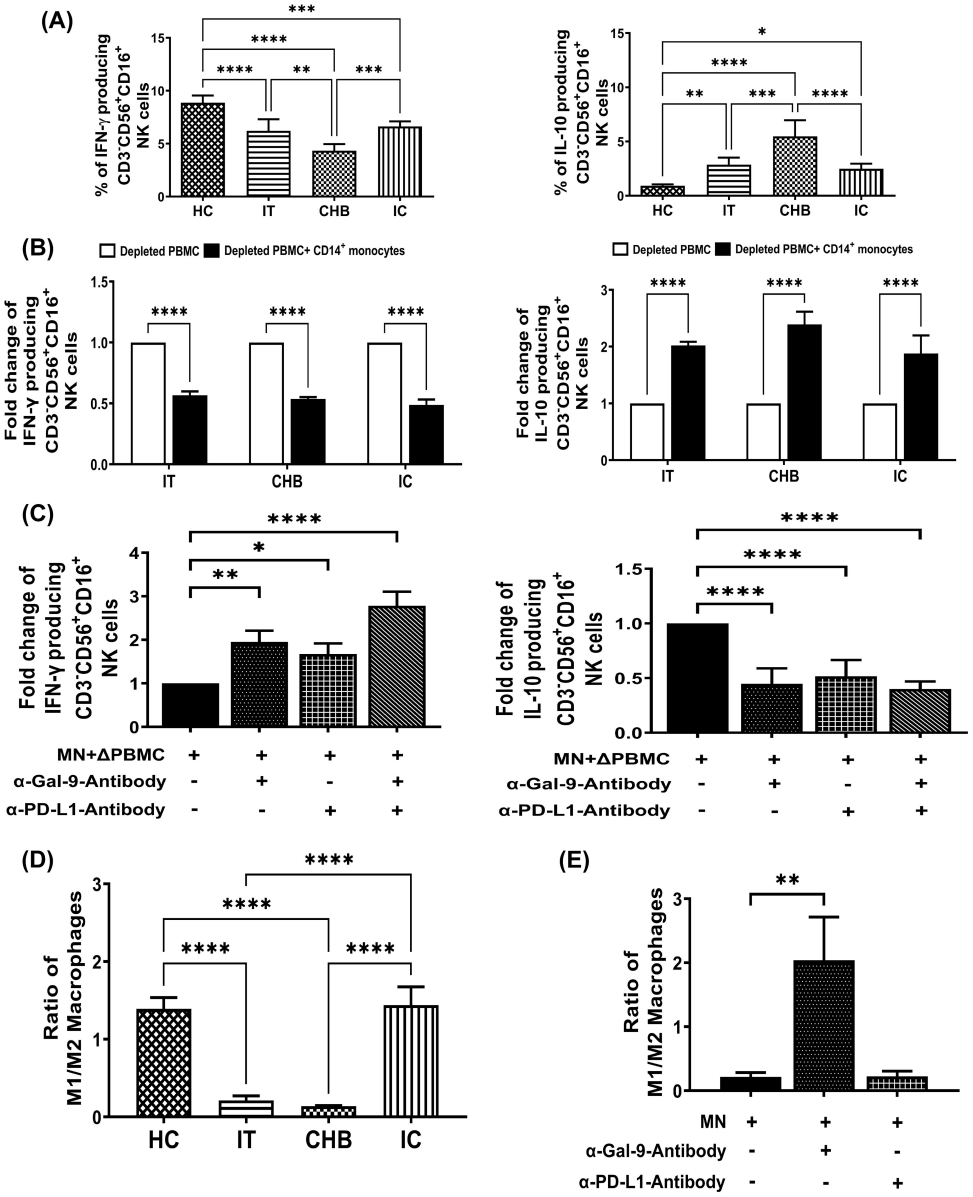
Monocyte-derived Gal-9 and PD-L1 alter NK cell functions and M1-/M2-macrophage ratio during CHI. **(A)** Bar diagrams depicting percentages of CD3^-^CD56^+^CD16^+^ NK cells expressing cytokines IFN-γ and IL-10 in immune-tolerant (IT) (n=10), chronic hepatitis B (CHB) (n=10) and inactive carriers (IC) (n=10) and healthy controls (HC) (n=10) **(B)** Grouped bar diagram representing fold changes in the frequencies of CD3^-^CD56^+^CD16^+^IFN-γ^+^ and CD3^-^CD56^+^CD16^+^IL-10^+^ NK-cells following co-culture of sorted CD14^+^ monocytes with rIL-12/rIL-18-stimulated autologous monocyte-depleted PBMCs derived from IT, CHB and IC. **(C)** Monocytes (MN) sorted from CHB were co-cultured with autologous monocyte depleted PBMCs (ΔPBMC) stimulated with rIL-12 and rIL-18 in absence or presence of anti-Gal-9 (α-Gal-9) and anti-PD-L1-antibody (α-PD-L1) and percentages of IFN-γ and IL-10 expressing CD3^-^CD56^+^CD16^+^ NK cells were determined. **(D)** Bar diagrams demonstrating ratio of *in vitro* differentiated M1- and M2-macrophages from monocytes of IT (n=10), CHB (n=10), IC (n=10) and HC (n=10). **(E)** Grouped bar diagram representing changes in the ratio of M1- and M2- macrophages following differentiation of purified monocytes (MN) with or without neutralizing antibodies against Gal-9 (α-Gal-9) and PD-L1 (α-PD-L1). Comparisons between groups were performed using the one-way ANOVA with p values adjusted by the Tukey’s multiple comparison test in **(A)** and **(D)**. t Tests were performed for comparing paired groups and Mean ± SD of three individual set of experiments in each study group is given in **(B, C, E)**. *p < 0.05, **p < 0.01, ***p < 0.001, ****p < 0.0001.

NK-cells play a key role in antiviral immunity through secretion of cytokines, including their signature cytokine, IFN-γ (13). However, NK-cells displayed aberrant cytokine profile in CHB (14, 15) and we explored whether monocytes via their ICMs elicit these defects. Our results indicated that IFN-γ^+^ NK-cells were significantly decreased in IT, CHB and IC as compared to HC but the decline was most prominent in CHB (**Figure 4A**). Conversely, IL-10^+^ NK-cells that were barely perceptible in HC, were increasingly prevalent in IT, IC and CHB (**Figure 4A**). Moreover, in CHB, IT and IC, exposure to autologous purified monocytes led to decrease in IFN-γ and a considerable augmentation in IL-10 production by NK-cells (**Figure 4B**). Further, single and combined blockade of Gal-9 and PD-L1 markedly improved IFN-γ and suppressed IL-10 production by NK-cells, confirming involvement of these ICMs in regulating the cytokine production by NK-cells in chronically HBV-infected patients (**Figure 4C**, **Supplementary Figure S8**).

### Gal-9-expressing monocytes preferentially differentiate into M2-macrophages in CHI

Monocytes traffic to sites of infection and depending on microenvironment, differentiate into either classically-activated M1-macrophages or alternatively-activated M2-macrophages with distinct phenotype and function (16). We investigated whether elevated Gal-9 and PD-L1 on monocytes affect monocyte to macrophage differentiation during CHI. For this, monocytes from different study subjects were differentiated *in vitro* into M1- or M2-macrophages and M1-/M2-macrophage ratio was evaluated using TNF-α^+^ and IL-10^+^ cell proportions respectively among CD14^+^HLA-DR^+^CD68^+^ macrophages. M1/M2 ratio was found to be reduced in IT and CHB patients than IC or HC (**Figure 4D**). Moreover, anti-Gal-9 treatment of CHB monocytes caused an enhanced switching of differentiated macrophages towards the M1-phenotype with consequent increase in M1/M2 ratio whereas, no change was detected upon incubation with anti-PD-L1-antibody (**Figure 4E**), highlighting the participation of monocytic Gal-9 in M2-macrophage polarization.

### Intrahepatic accumulation of Gal-9- and PD-L1-expressing monocytes in CHB patients

**Figure 5:**
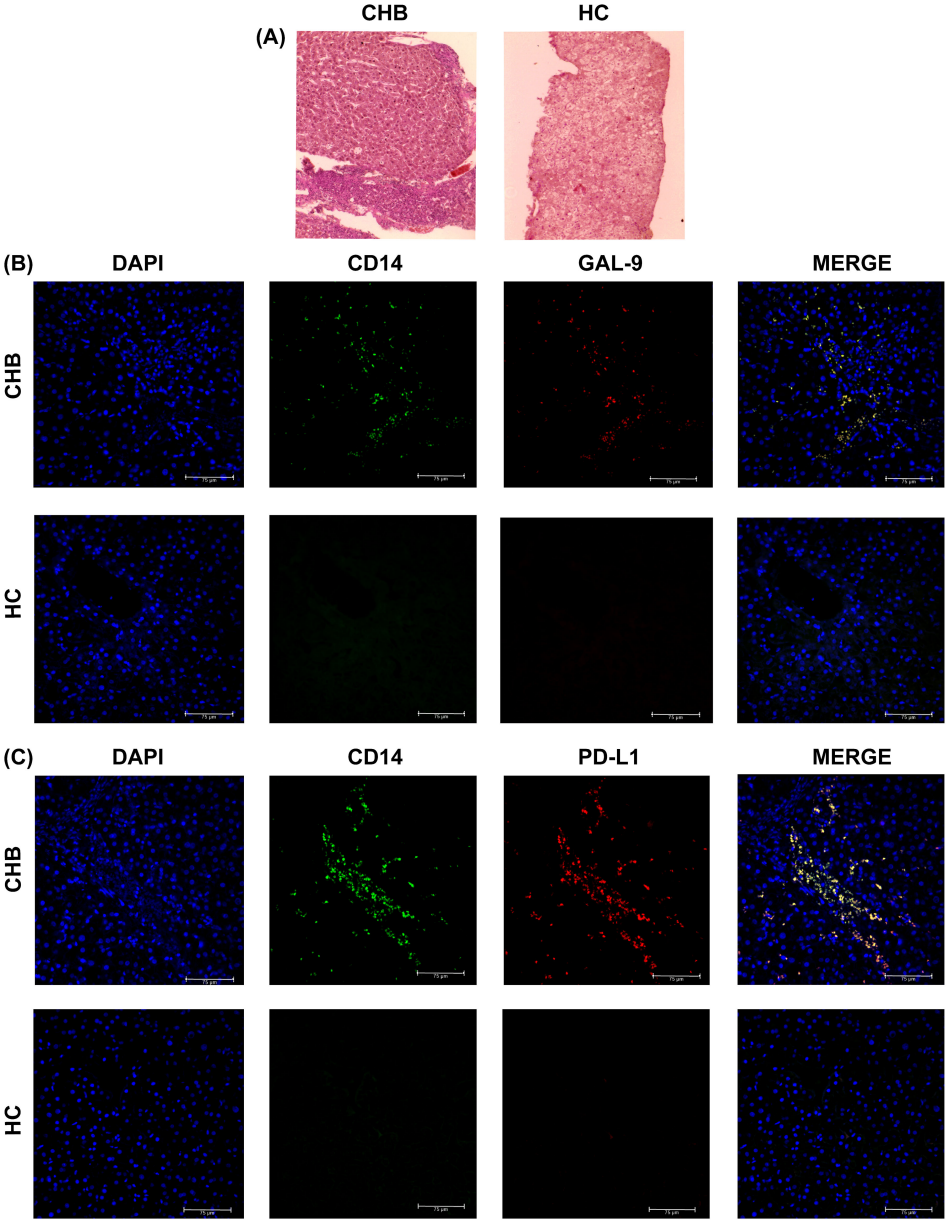
Intrahepatic incidence of Gal-9- and PD-L1-expressing monocytes in CHB and HC: **(A)** Haematoxylin and Eosin staining of liver biopsy tissue sections of selected CHB patient and Healthy individual (HC). Intrahepatic incidence of **(B)** DAPI^+^ (blue), CD14^+^ (green), Gal-9^+^ (red), CD14^+^Gal-9^+^ (Yellow) and **(C)** DAPI^+^ (blue), CD14^+^ (green), PD-L1^+^ (red), CD14^+^PD-L1^+^ (Yellow) cells in liver biopsy tissue sections of CHB patient and HC.

We studied the frequencies of CD14^+^Gal-9^+^- and CD14^+^PD-L1^+^-monocytes in selected liver-biopsy-sections of CHB patients and HC by immunohistochemical staining. Liver histology of CHB displayed prominent lymphocyte-predominant lobular and portal inflammation (**Figure 5A**) and an enrichment of monocytes expressing Gal-9 and PD-L1 was observed in hepatic compartment of CHB in comparison to HC (**Figures 5B, C**).

### Monocytic Gal-9/PD-L1-expression remained unaltered in TDF-treated CHB patients

**Figure 6:**
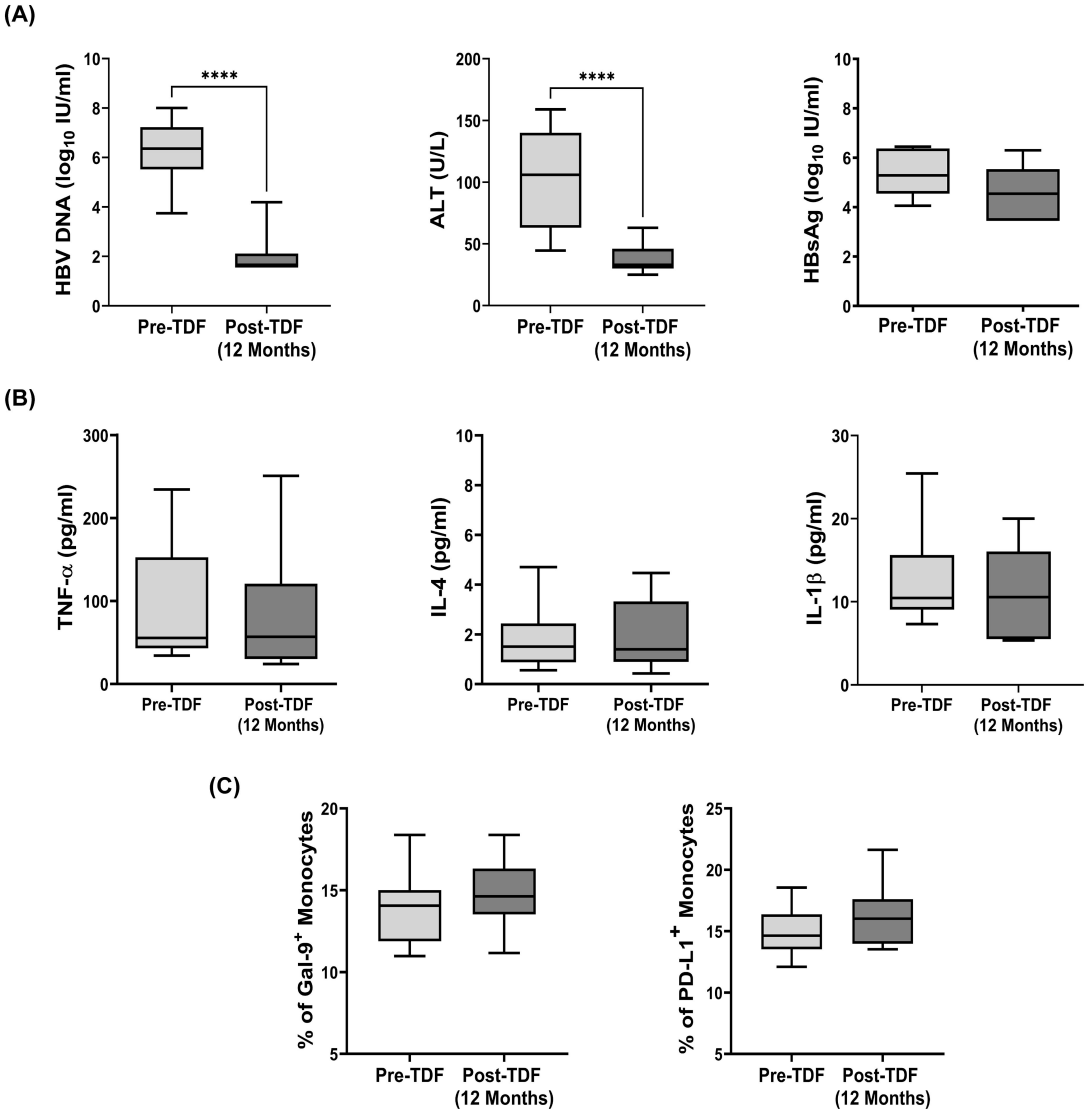
Expression of Gal-9 and PD-L1 on monocytes and viral/clinical parameters in Tenofovir treated patients: **(A)** HBV DNA, serum ALT, serum levels of HBsAg, **(B)** TNF-α, IL-4, IL-1β and **(C)** frequency of Gal-9^+^ and PD-L1^+^ total monocytes in CHB patients (n=15) before initiation (pre-TDF) and after one year of antiviral Tenofovir therapy (post-TDF). Paired t tests were performed for comparing paired groups in **(A)** to **(C)**. ****p < 0.0001. Mean ± SD are presented.

TDF is recommended as first-line monotherapy for CHB patients (17) and we tested the effect of TDF on monocytic Gal-9 and PD-L1-expression in 15 CHB patients that included 8 EP-CHB and 7 EN-CHB. We observed that all CHB patients achieved significant reduction of HBV-DNA and normalization of serum ALT after 1 year of therapy (**Figure 6A**), but no significant difference was perceived in the proportion of Gal-9-expressing and PD-L1-expressing monocytes between pre-treatment and post-treatment time points (**Figure 6C**). In parallel, the serum levels of HBsAg, TNF-α, IL-4, and IL-1β, which are important for inducing the expression of monocytic Gal-9 and PD-L1 remained similar to the baseline values in these patients following 1 year of TDF treatment (**Figures 6A, B**).

## Discussion

The present study highlights the previously unappreciated importance of monocyte-derived ICMs in shaping the immune response during CHI. We uncovered heterogeneous ICM-expression pattern on monocytes in different phases of CHI, which was controlled by HBsAg and systemic cytokines and demonstrated that these ICMs influence the interaction of monocytes with T-, B- and NK-cells and monocyte to macrophage differentiation by exerting distinct regulatory effects. This knowledge would add to the ongoing efforts of understanding the molecular basis of failed immune response in chronically HBV-infected patients and pave way for the rational design of novel immunotherapeutic strategies aimed at reversing the defects.

High expression of Gal-9 and PD-L1 was found to be the hallmark of monocytes in EP-/EN-CHB patients while monocytes from IT displayed heightened expression of only Gal-9 and that of IC predominantly expressed PD-L1 as compared with HC. We established a causal influence of high HBsAg and TNF-α concentration on Gal-9-expression by monocytes while elevated levels of TNF-α, IL-4 and IL-1β could individually contribute to the induction of monocytic PD-L1. Our findings however, contradict a previous study involving Chinese patients that depicted HBsAg-mediated induction of PD-L1-expression in monocytes (15). Studies in patients with systemic lupus erythematosus had shown that TNF-α boost PD-L1-expression in monocytes (18) and this upregulation is likely to occur through TNF-α-mediated activation of NF-κB and ERK1/2 pathways as deciphered from studies in human colon cancer HCT116 cells (19). Additionally, TNF-α-dependent enhancement of Gal-9-expression had been described earlier in primary astrocytes via TNFR1/JNK/c-Jun signalling pathway (20). Further, the combined IL-4 and TNF-α treatment had been found to cause synergistic augmentation in PD-L1-expression in renal carcinoma cells (21) while the inductive effect of IL-1β on PD-L1-expression had been demonstrated in lung cancer and glioma cell lines (22).

We next investigated the diverse effects exerted by monocyte-associated Gal-9 and PD-L1 during the cross-talk of monocytes with other immune-cells in various clinical stages of CHI. We demonstrated that Gal-9- and PD-L1-expressing monocytes are intimately involved in undermining the HBV-specific T-/B-cell and NK-cell functions in CHI and that each of these two ICMs has distinct immune-modulating role. While monocytic Gal-9 could exclusively favour the generation of Treg, atMB-cells and M2-macrophages, PD-L1 was solely responsible for attenuating the production of important cytokines IL-2/IFN-γ or IL-6 by virus-specific T- and B-cells. Both Gal-9 and PD-L1 however, could enhance IL-10 production by HBV-specific T-cells and NK-cells, diminish IFN-γ secretion by NK-cells and promote Breg generation. Our findings resonate with the result of an earlier study that showed HBV-induced PD-L1 on monocytes promoted the generation of IL-10^+^ regulatory NK-cells (15). The critical role of PD-L1 expressed by tumor-infiltrating dendritic cells in limiting T-cell responses had been illustrated in a recent study (23).

**Figure 7:**
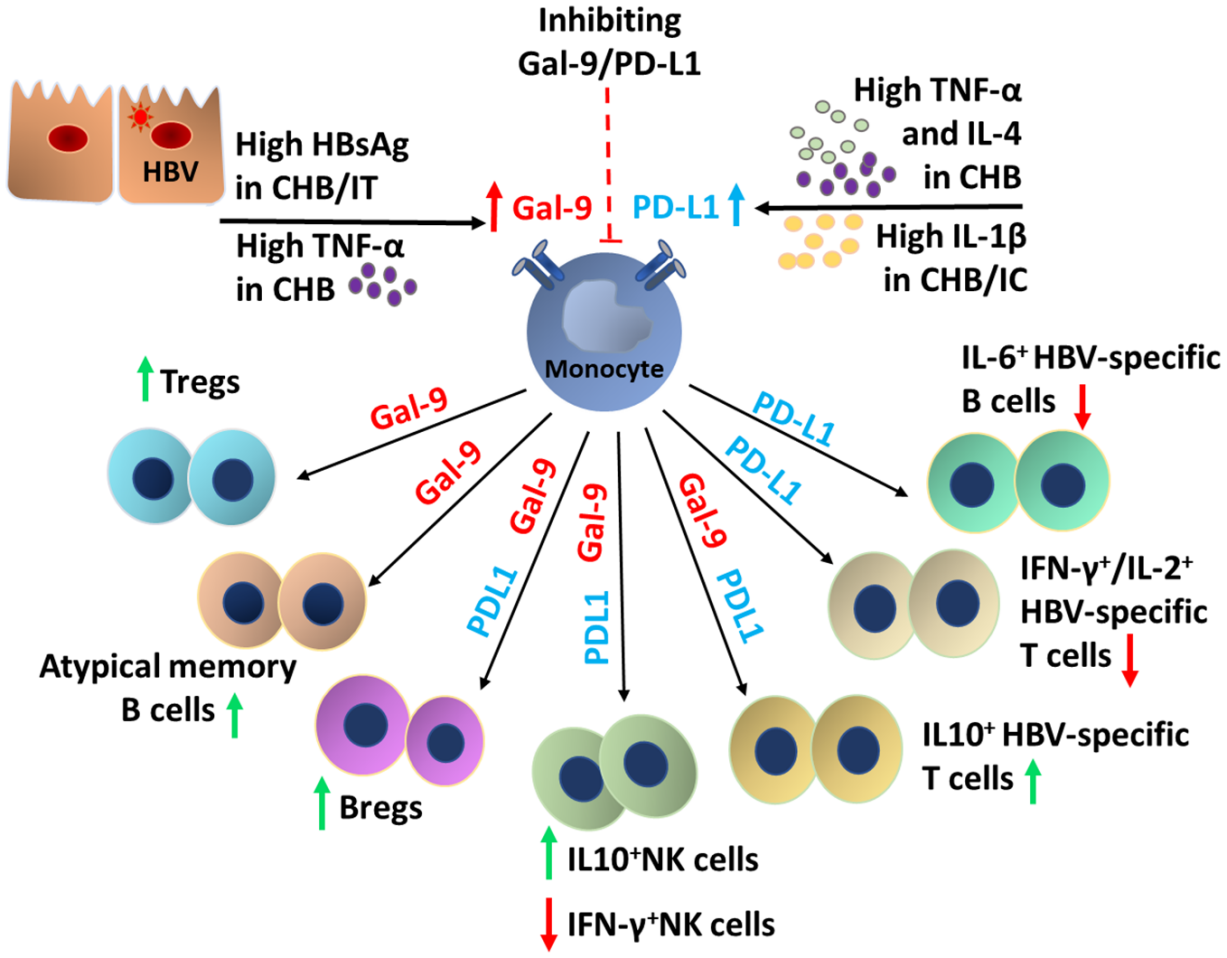
Schematic overview of the factors responsible for the induction of monocytic Gal-9 and PD-L1 and the broad spectrum of functional alterations in the T-cell, B-cell, NK cell and macrophage mediated by them during chronic HBV infection.

Taken together, as summarized in **Figure 7**, our study suggests that in IT, HBsAg stimulated the Gal-9-expression on monocytes, which in turn contributes to the induction of Treg, Breg, atMB-cells, IL-10-expressing T-/NK-cells and M2-macrophages but it did not interfere with anti-viral cytokine production by virus-specific T-/B-cells. In CHB, the synergistic effects of high HBsAg concentration accompanied by elevated TNF-α, IL-4 and IL-1β upregulated both Gal-9 and PD-L1 on monocytes that conveyed multifaceted impacts by not only triggering the Gal-9-dependent immunological changes as mentioned before but also PD-L1-induced attenuation of anti-viral cytokine release by HBV-specific T-/B-cells and NK-cells, thereby severely impairing the host immune response. On the contrary, in IC, high IL-1β level enhanced PD-L1-expression on monocytes, albeit to a lesser extent than that observed in CHB. Consequently, PD-L1-elicited insufficiencies in T-/B-/NK-cell responses though present in IC, was less profound than CHB.

We further demonstrated that one-year of TDF-therapy could effectively control HBV-replication in CHB patients but fail to reduce the frequency of Gal-9^+^/PD-L1^+^-monocytes. Our findings are in agreement to that of Ferrando-Martinez et al., who reported that monocytic PD-L1 expression in HBV-infected patients was not restored to homeostatic levels after successful antiviral therapy with tenofovir, entecavir or lamivudine (24). We also noted that treatment with TDF could not lower the level of serum HBsAg that is usually produced in large excess over complete-virions and this in turn could promote Gal-9 expression on monocytes. Moreover, the serum cytokines, TNF-α, IL-4 and IL-1β continued to remain high even after TDF-therapy, which might persuade high PD-L1-expression. The sustained persistence of high monocytic ICM-expression even after anti-viral therapy may thus represent a pivotal risk factor for advanced liver diseases such as HCC. Previous studies had also shown that Gal-9 expression on Kupffer cells of HBV-associated HCC tumor tissues was closely correlated with poor prognosis (25). Circulating PD-1/PD-L1 expression had also been found to be associated with severity of diseases in HCC patients (26, 27). The results presented here establish Gal-9- and PD-L1-expressing monocytes as critical gatekeepers that prevent effective antiviral immune responses in CHI. Modulation of monocytic Gal-9/PD-L1-expression or blocking the interaction between these ICMs and their cognate receptors with immune checkpoint inhibitors may thus represent a potential approach for reinvigoration of immune response in chronically HBV-infected patients, thereby facilitating viral clearance and cure of HBV-induced liver diseases. However, close and careful monitoring is required before use of these therapeutic strategies in CHI in order to balance the possible clinical benefits and the risks associated with these therapies such as viral reactivation or immune-related adverse effects.

## Data Availability

The original contributions presented in the study are included in the article/**Supplementary Material**. Further inquiries can be directed to the corresponding author.
