# Supplementary material for: Monocyte-derived Galectin-9 and PD-L1 differentially impair adaptive and innate immune response in chronic HBV infection and their expression remain unaltered after antiviral therapy
Source: Front Immunol. 2024 Oct 9;15:1474853. doi: 10.3389/fimmu.2024.1474853 (PMC11496065; doi:10.3389/fimmu.2024.1474853)
Supplement: Supplementary file 1 [file DataSheet1.docx]

**Supplementary Information**

**Monocyte-derived Galectin-9 and PD-L1 differentially impair adaptive and innate immune response in chronic HBV infection and their expression remain unaltered after antiviral therapy**

Debangana Dey^1^, Satabdi Biswas^1^, Sourina Pal^1^, Sarthak Nandi^1^, Najma Khatun^1^, Rambha Jha^1^, Bidhan Chandra Chakraborty^2^, Ayana Baidya^1^, Ranajoy Ghosh^3^, Soma Banerjee^1^, SK Mahiuddin Ahammed^4^, Abhijit Chowdhury^4^ and Simanti Datta^1^*

^1^Centre for Liver Research, School of Digestive and Liver Diseases, Institute of Post Graduate Medical Education and Research, Kolkata, India

^2^Multidisciplinary Research Unit, Institute of Post Graduate Medical Education and Research, Kolkata, India

^3^Division of Pathology, School of Digestive and Liver Diseases, Institute of Postgraduate Medical Education and Research, Kolkata, India

^4^Department of Hepatology, School of Digestive and Liver Diseases, Institute of Post Graduate Medical Education and Research, Kolkata, India

Medical Education and Research, Kolkata, India

**Addresses correspondence to**

Prof. Simanti Datta,

Centre for Liver Research, School of Digestive and Liver Diseases,

Institute of Post Graduate Medical Education and Research,

244, A.J. C. Bose Road, Kolkata-700020, India.

Tel- (91)-(033)-2223 5435; Fax- (91)-(033)-2223 6383

E-mail- [seemdatt@gmail.com](mailto:seemdatt@gmail.com)

**Supplementary Table S1** Demographic, biochemical and virological characteristics of HBV-infected individuals belonging to different clinical stages and healthy controls

| **Characteristics** | **HC**  (N=20) | **RESOLVED**  **ACUTE**  (N=10) | **IT**  (N=10) | **EP-CHB**  (N=20) | **IC**  (N=20) | **EN-CHB**  (N=21) |
| --- | --- | --- | --- | --- | --- | --- |
| **Age, years Median (Range)** | 30  (25-45) | 34  (20-51) | 18  (8-22) | 32  (25-62) | 35  (27-52) | 39  (31-67) |
| **Sex**  **(Male:Female)** | 8:12 | 7:3 | 6:4 | 15:5 | 7:13 | 17:4 |
| **HBeAg Status** | - | - | Positive | Positive | Negative | Negative |
| **ALT(IU/L) Median (Range)** | 17  (12-26) | 29  (15-39) | 30  (21-38) | 106  (50-236) | 23  (12-35) | 111  (48-145) |
| **HBV DNA (copies/ml) Median (Range)** | - | - | 1.7X10^8^  (1.2X10^7^-2X10^8^) | 1.4X10^7^  (5.5X10^4^-1.7X10^8^) | 250  (128-700) | 3.3X10^4^  (1.8X10^4^-1X10^8^) |

IT, Immunotolerant; EP-CHB, HBeAg-positive chronic hepatitis B; IC, Inactive carriers; EN-CHB, HBeAg-negative chronic hepatitis B; HC, Healthy controls, RESOLVED ACUTE, Acutely HBV infected patients who resolved infection; ALT, alanine aminotransferase; IU, international unit;

**Supplementary Table S2** List of Antibodies

| SL No. | Reagent Name | Origin/Clone | Cat No. | Company name |
| --- | --- | --- | --- | --- |
| 1 | Antihuman-CD14-FITC | M5E2 | 555397 | BD Biosciences |
| 2 | Antihuman-HLA-DR-BV421 | L243 | 307636 | BioLegend Inc. |
| 3 | Antihuman-CD16-PECY7 | 3G8 | 557744 | BD biosciences |
| 4 | Antihuman-Galectin-9-PerCP | 9M1-3 | 348910 | BioLegend Inc. |
| 5 | Antihuman-PD-L1-PE | 29E.2A3 | 329705 | BioLegend Inc. |
| 6 | Antihuman-PD-L1-APC | M1H3 | 374513 | BioLegend Inc. |
| 7 | Antihuman-CTLA-4-APC | BNI3 | 555855 | BD Biosciences |
| 8 | Antihuman-CD68-FITC | Y1/82A | 333806 | BioLegend Inc. |
| 9 | Antihuman-CD4-FITC | RPA-T4 | 30056 | BioLegend Inc. |
| 10 | Antihuman-CD25-PECY7 | M-A251 | 561405 | BD Biosciences |
| 11 | Antihuman-FoxP3-PE | 259D/C7 | 560046 | BD Biosciences |
| 12 | Purified anti-CD3 antibody | HIT3a | 300314 | BioLegend Inc. |
| 13 | Purified anti-CD28 antibody | CD28.2 | 302914 | BioLegend Inc. |
| 14 | Antihuman-CD8-PERCP | SK1 | 344708 | BioLegend Inc. |
| 15 | Antihuman- IFN-γ –PE | B27 | 506506 | BioLegend Inc. |
| 16 | Antihuman- IFN-γ –BV421 | 4S.B3 | 564791 | BD Biosciences |
| 17 | Antihuman- TNF-α –APC | MAb11 | 551384 | BD Biosciences |
| 18 | Antihuman- IL-2 –PE | MQ1-17H12 | 500307 | BioLegend Inc. |
| 19 | Antihuman- IL-10 –APC | JES3-19F1 | 554707 | BD Biosciences |
| 20 | Antihuman- IL-10 –PE | JES3-19F1 | 559330 | BD Biosciences |
| 21 | Antihuman- IL-6 –PE | MQ2-6A3 | 559331 | BD Biosciences |
| 22 | Antihuman-CD56-PECY7 | B159 | 557747 | BD Biosciences |
| 23 | Antihuman-CD3-PERCP | SP34-2 | 552851 | BD Biosciences |
| 24 | Antihuman-CD16 BV421 | B73.1 | 360724 | BioLegend Inc. |
| 25 | Antihuman-CD19-FITC | HIB19 | 555412 | BD Biosciences |
| 26 | Antihuman-CD21-BV421 | B-ly4 | 566260 | BD Biosciences |
| 27 | Antihuman-CD27-PECY7 | M-T271 | 560609 | BD Biosciences |
| 28 | Recombinant Human IL-12 | - | 573004 | BioLegend Inc. |
| 29 | Recombinant Human IL-18 | - | 592104 | BioLegend Inc. |
| 30 | Purified-Antihuman -Galectin-9 | 9M1-3 | 348902 | BioLegend Inc. |
| 31 | Purified-Antihuman-CD274 (PD-L1) | 29E.2A3 | 329716 | BioLegend Inc. |

**Supplementary Figure S1**

**
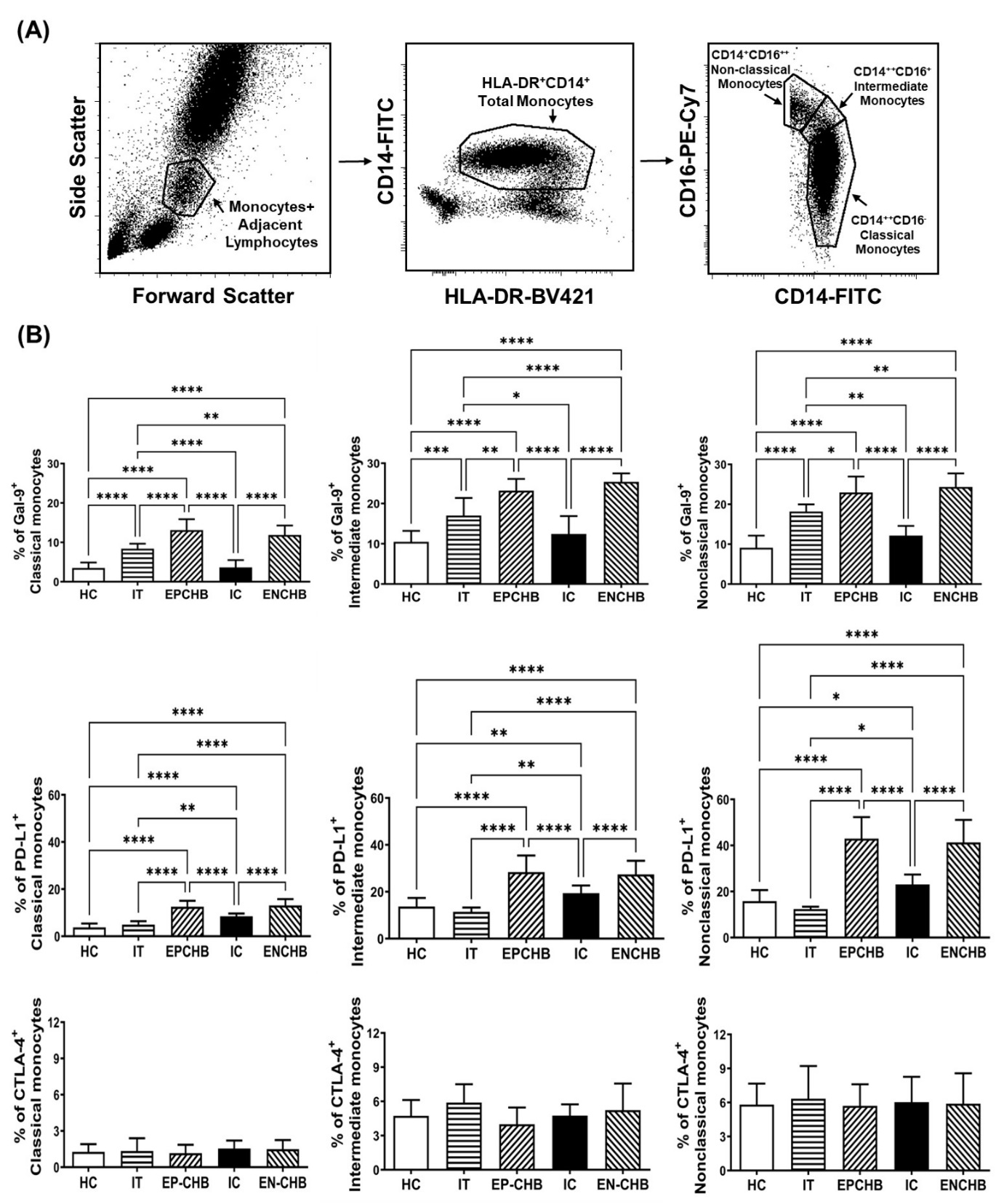
**

**Figure S1 (A)** Sequential gating strategy for identification of HLADR^+^CD14^+^ total, HLA-DR^+^CD14^++^CD16^-^ classical, HLA-DR^+^CD14^++^CD16^+^ intermediate and HLA-DR^+^CD14^+^CD16^++^ non-classical monocytes from whole blood using immunophenotyping followed by flow cytometry in all the study groups. On total monocytes and each subset, the expression of Gal-9, PD-L1 or CTLA-4 was determined. **(B)** Bar diagrams demonstrating pooled data of percentages of classical, intermediate and non-classical monocytes expressing Gal-9 (upper panel), PD-L1(middle panel) and CTLA-4 (lower panel) in Immune-tolerant (IT), HBeAg-positive CHB (EP-CHB), Inactive carriers (IC), HBeAg-negative CHB (EN-CHB) and healthy controls (HC). Statistical significance was assessed by one way ANOVA test followed by Tukey's Multiple Comparison Test. (**P* < 0.05, ***P* < 0.01, ****P* < 0.001 and *****P* < 0.0001).

**Supplementary Figure S2**


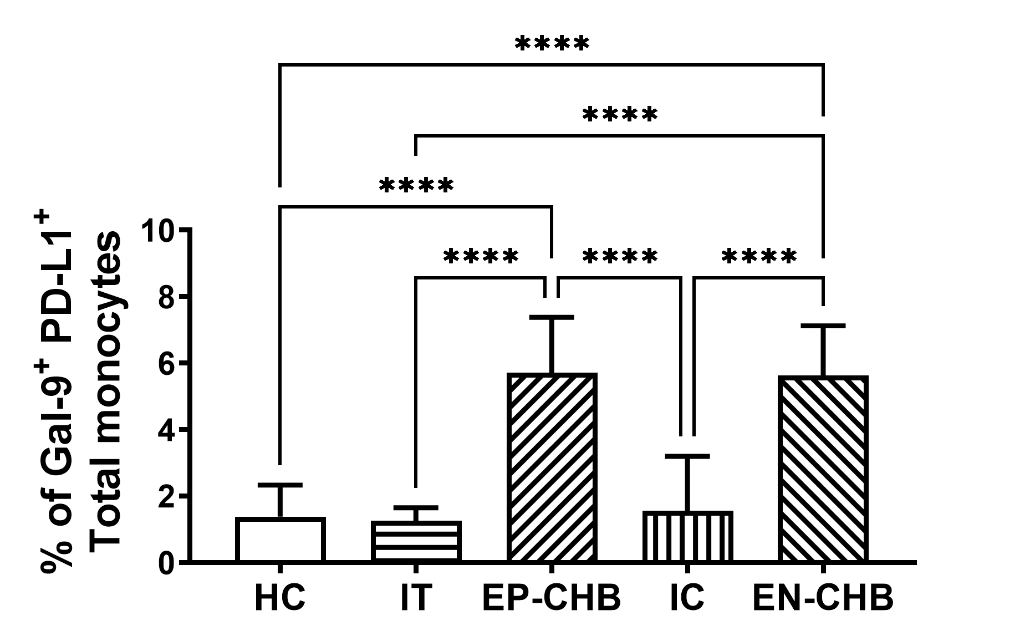


**Figure S2:** Bar diagrams demonstrating percentages of Gal-9 and PD-L1 dual expressing total monocytes in healthy controls (HC), immune-tolerant (IT), HBeAg-positive CHB (EP-CHB), inactive carriers (IC) and HBeAg-negative CHB (EN-CHB). Statistical significance was assessed by one way ANOVA test followed by Tukey's Multiple Comparison Test. (**P* < 0.05, ***P* < 0.01, ****P* < 0.001 and *****P* < 0.0001).

**Supplementary Figure S3**


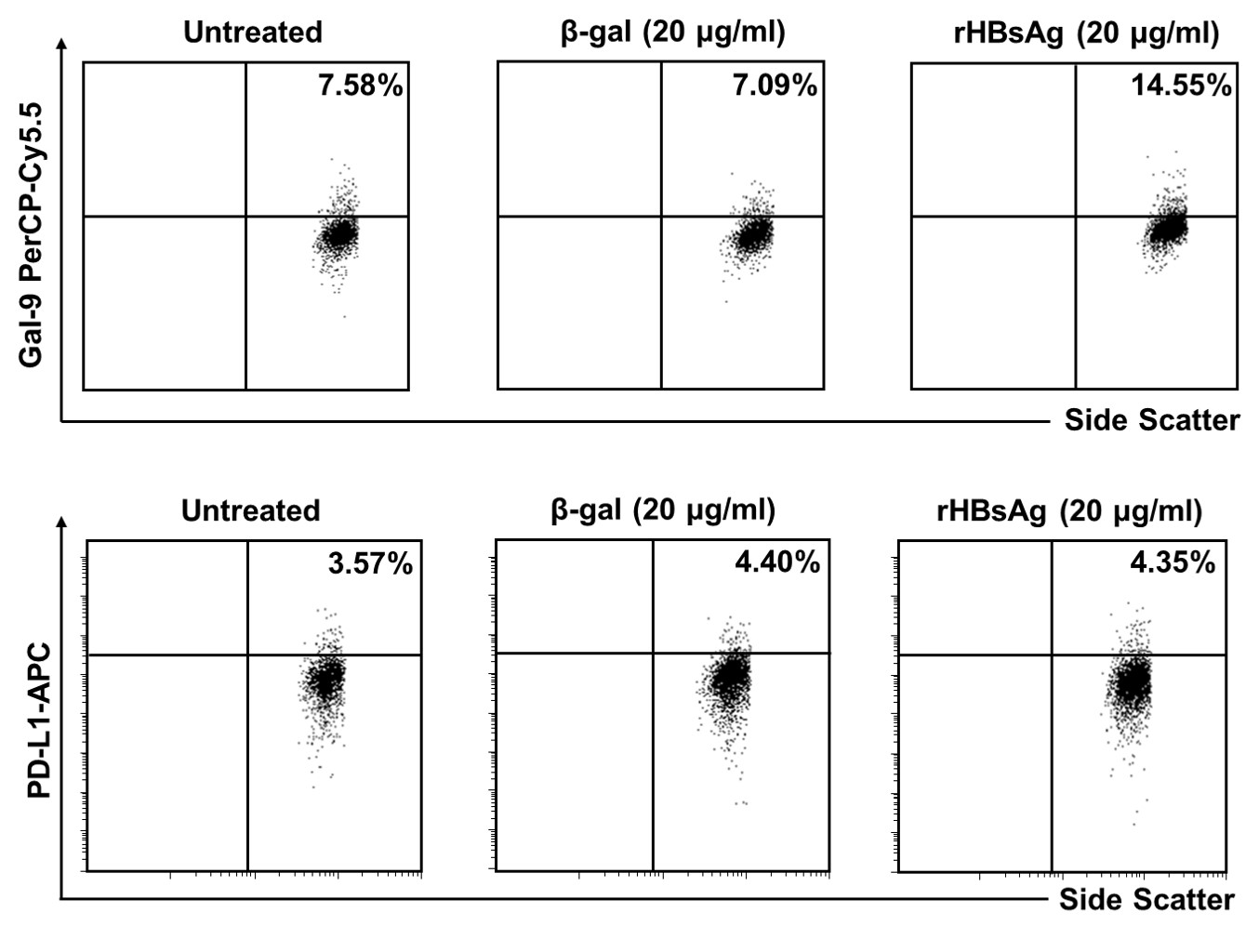


**Figure S3** Representative FACS plots displaying the expression of Gal-9 (upper panel) and PD-L1 (lower panel) on sorted CD14^+^ monocytes of a healthy individual following treatment with β‐galactosidase (β‐gal) (20μg/ml) and recombinant Hepatitis B surface antigen (rHBsAg) (20μg/ml).

**Supplementary Figure S4**

**
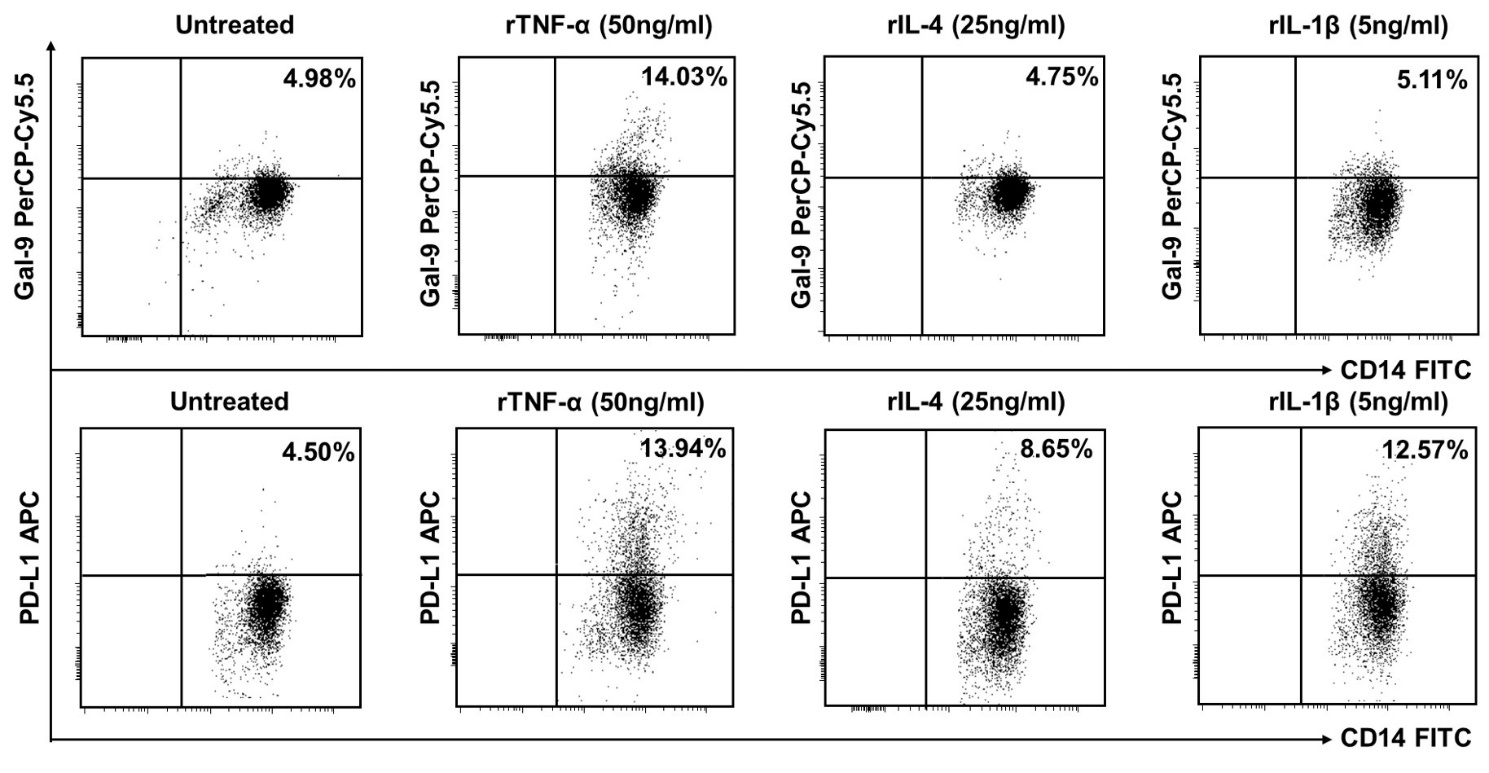
**

**Figure S4** Representative FACS plots displaying the frequencies of monocytes of a healthy control (HC) expressing Gal-9 (upper panel) and PD-L1 (lower panel) following treatment of PBMC with recombinant (r) TNF-α (50ng/ml), rIL-4 (25ng/ml) and rIL-1β (5ng/ml).

**Supplementary Figure S5**

**
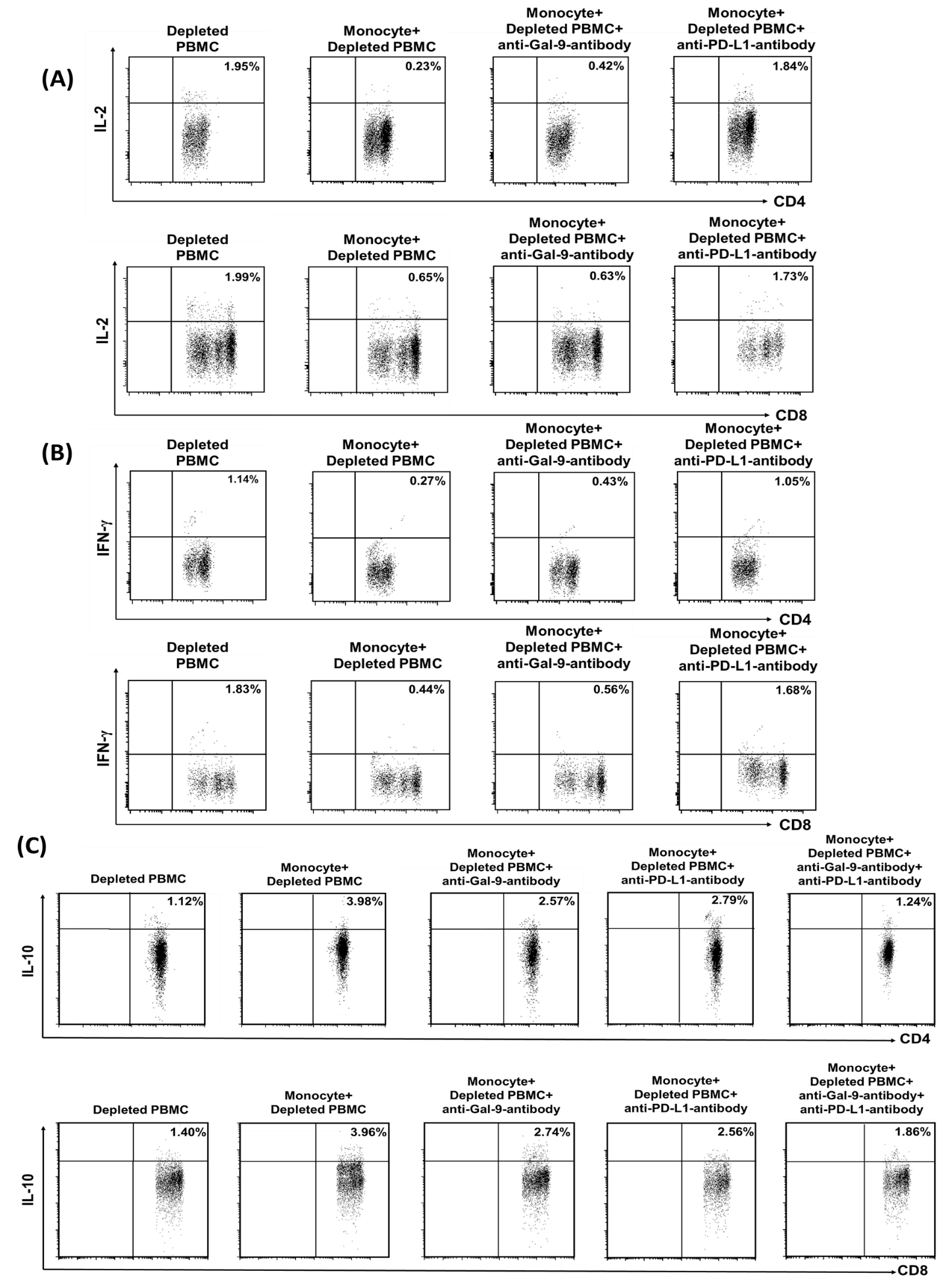
**

**Figure S5** Representative FACS dot plots depicting frequencies of HBV-specific CD4^+^- and CD8^+^-T-cells expressing (A) IL-2, (B) IFN-γ and (C) IL-10 following co-culture of sorted monocytes with HBV core peptide-stimulated autologous monocyte-depleted PBMC in absence or presence of anti-Gal-9-antibody or/and anti-PD-L1-antibody.

**Supplementary Figure S6**

**
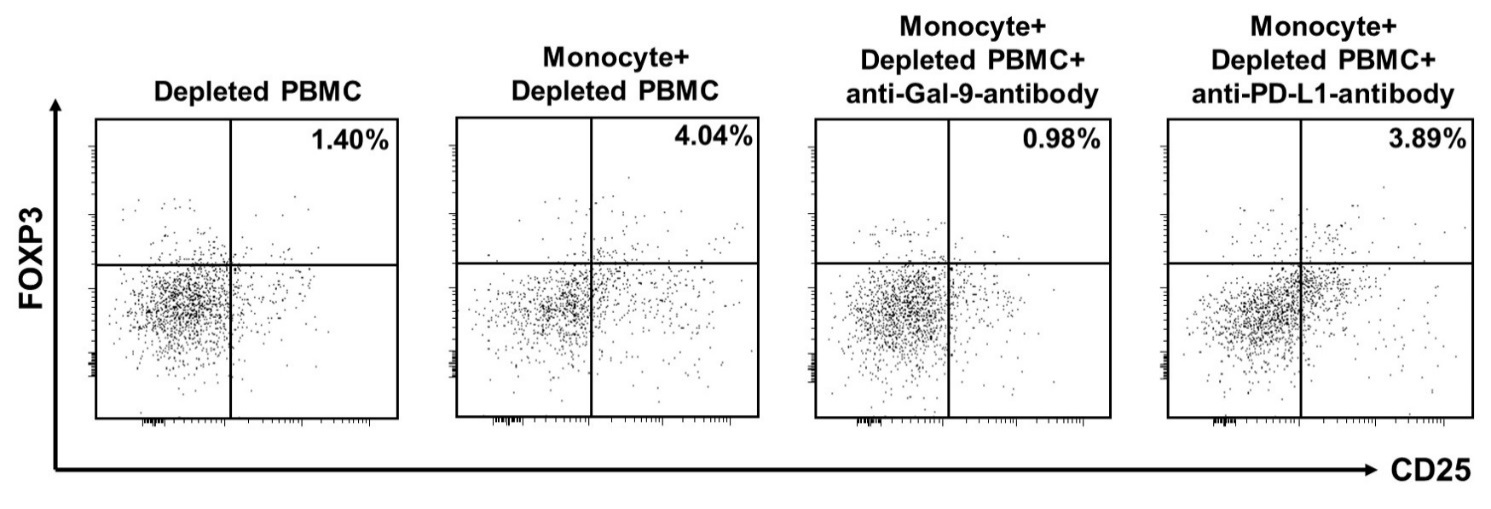
**

**Figure S6** Representative FACS dot plots showing frequencies of gated CD4^+^CD25^+^FOXP3^+^ Tregs following co-culture of sorted monocytes with anti-CD3/anti-CD28-stimulated autologous monocyte-depleted PBMC in absence or presence of anti-Gal-9-antibody or anti-PD-L1 antibody separately.

**Supplementary Figure S7**


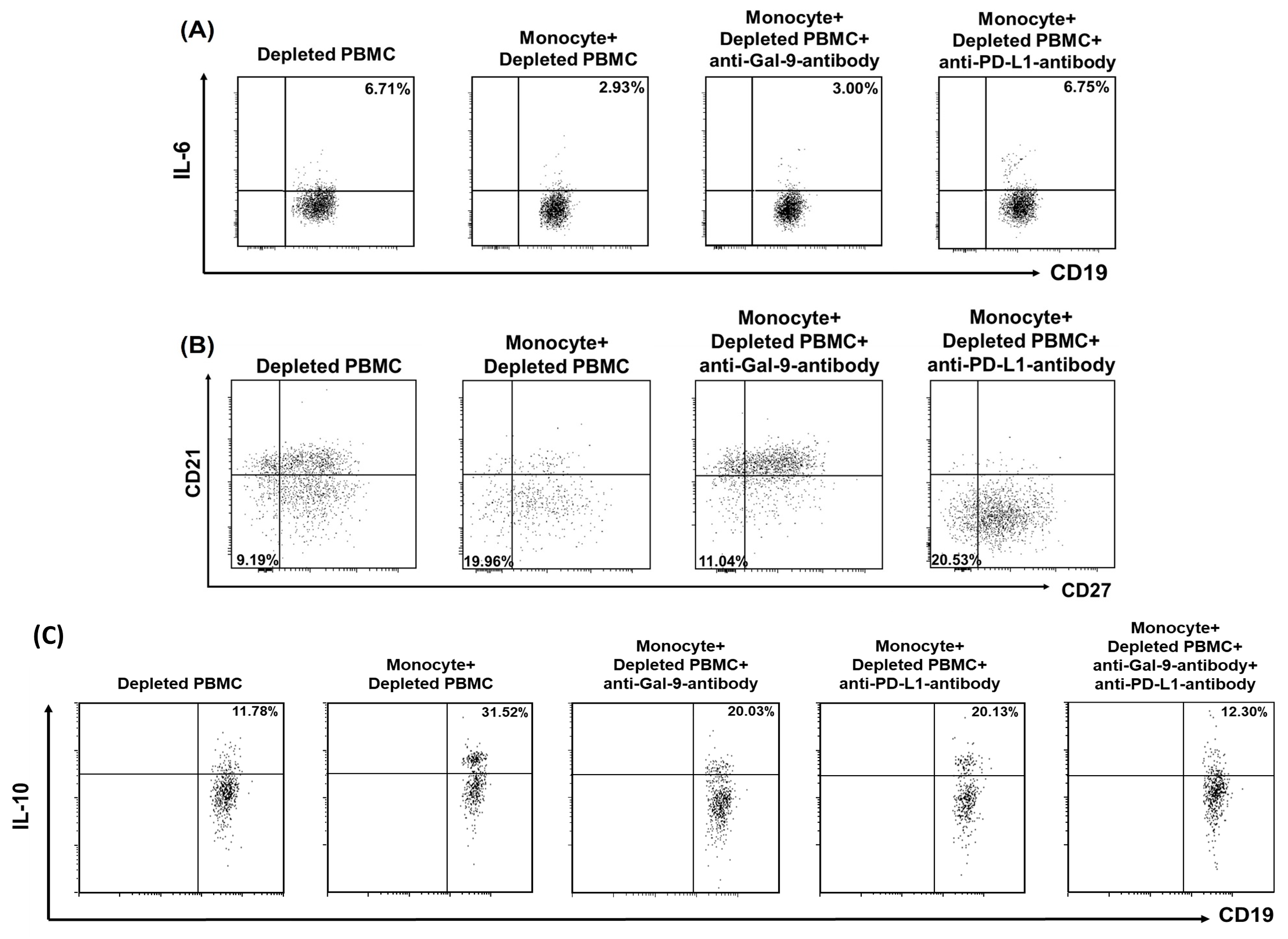


**Figure S7** Representative FACS dot plots depicting frequencies of gated (A) CD19^+^IL-6^+^ Hepatitis B core antigen (HBcAg)-specific B cells, (B) CD19^+^CD27^-^CD21^-^ atypical memory B cells and (C) CD19^+^IL-10^+^ regulatory B cells following co-culture of sorted monocytes with recombinant (r) HBcAg-stimulated or rCD40L-stimulated autologous monocyte-depleted PBMC as appropriate in absence or presence of anti-Gal-9-antibody or/and anti-PD-L1-antibody

**Supplementary Figure S8**

**
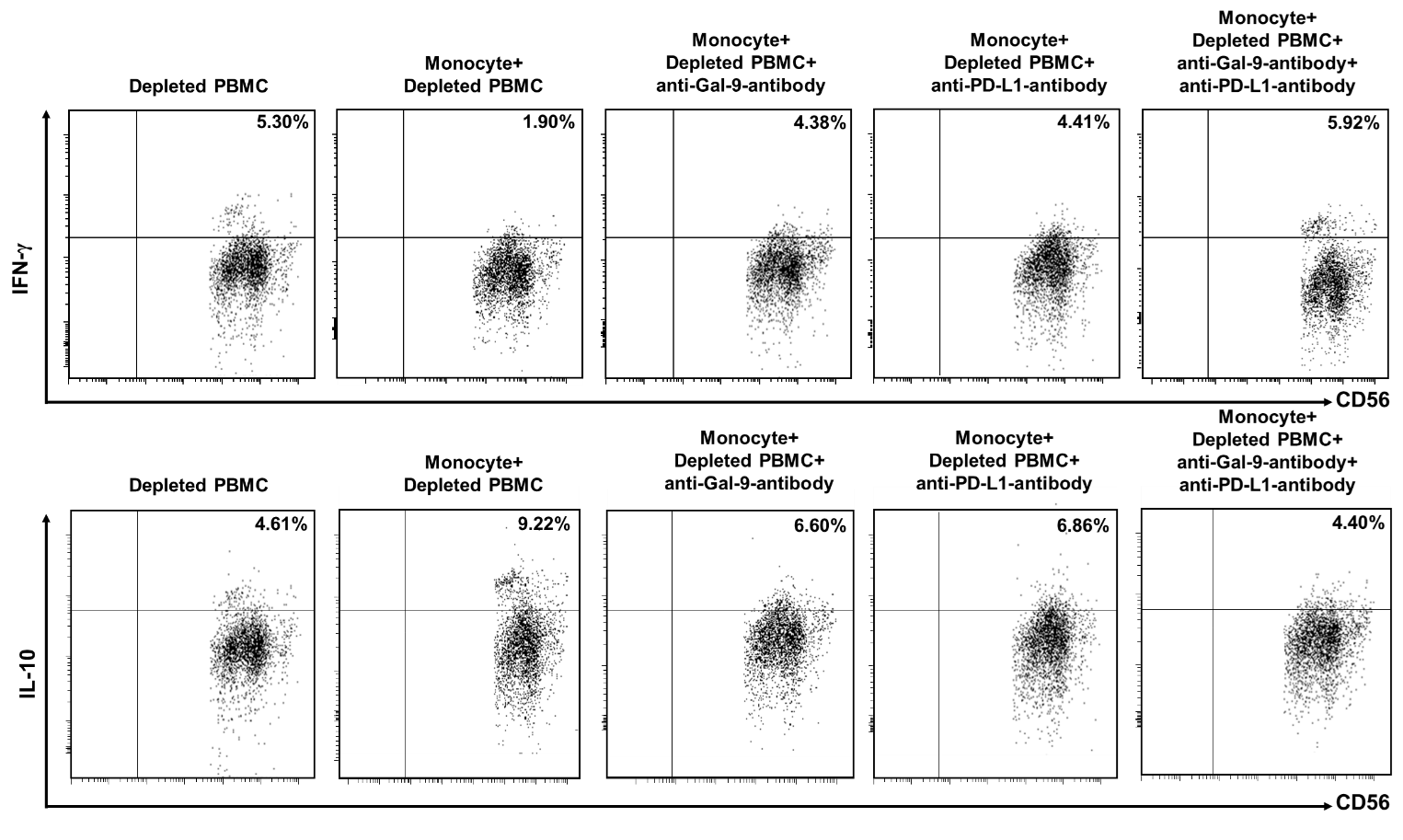
**

**Figure S8** Representative FACS dot plots depicting frequencies of gated CD3^-^CD56^+^CD16^+^ NK cells expressing IFN-γ (upper panel) and IL-10 (lower panel) following co-culture of monocytes with recombinant IL-12 and IL-18 stimulated autologous monocyte-depleted PBMC in absence or presence of anti-Gal-9-antibody or/and anti-PD-L1-antibody
